# Exploring the Therapeutic Potential of Phytochemicals in Alzheimer’s Disease: Focus on Polyphenols and Monoterpenes

**DOI:** 10.3389/fphar.2022.876614

**Published:** 2022-05-04

**Authors:** Ilaria Piccialli, Valentina Tedeschi, Lucia Caputo, Stefano D’Errico, Roselia Ciccone, Vincenzo De Feo, Agnese Secondo, Anna Pannaccione

**Affiliations:** ^1^ Division of Pharmacology, Department of Neuroscience, Reproductive and Dentistry Sciences, School of Medicine, University of Naples “Federico II”, Naples, Italy; ^2^ Department of Pharmacy, University of Salerno, Salerno, Italy; ^3^ Department of Pharmacy, University of Naples “Federico II”, Naples, Italy

**Keywords:** Alzheimer’s disease, phytochemicals, polyphenols, monoterpenes, multi-target therapy, neurodegeneration, neuroinflammation, amyloid-β aggregation

## Abstract

Alzheimer’s disease (AD) is a chronic, complex neurodegenerative disorder mainly characterized by the irreversible loss of memory and cognitive functions. Different hypotheses have been proposed thus far to explain the etiology of this devastating disorder, including those centered on the Amyloid-β (Aβ) peptide aggregation, Tau hyperphosphorylation, neuroinflammation and oxidative stress. Nonetheless, the therapeutic strategies conceived thus far to treat AD neurodegeneration have proven unsuccessful, probably due to the use of single-target drugs unable to arrest the progressive deterioration of brain functions. For this reason, the theoretical description of the AD etiology has recently switched from over-emphasizing a single deleterious process to considering AD neurodegeneration as the result of different pathogenic mechanisms and their interplay. Moreover, much relevance has recently been conferred to several comorbidities inducing insulin resistance and brain energy hypometabolism, including diabetes and obesity. As consequence, much interest is currently accorded in AD treatment to a multi-target approach interfering with different pathways at the same time, and to life-style interventions aimed at preventing the modifiable risk-factors strictly associated with aging. In this context, phytochemical compounds are emerging as an enormous source to draw on in the search for multi-target agents completing or assisting the traditional pharmacological medicine. Intriguingly, many plant-derived compounds have proven their efficacy in counteracting several pathogenic processes such as the Aβ aggregation, neuroinflammation, oxidative stress and insulin resistance. Many strategies have also been conceived to overcome the limitations of some promising phytochemicals related to their poor pharmacokinetic profiles, including nanotechnology and synthetic routes. Considering the emerging therapeutic potential of natural medicine, the aim of the present review is therefore to highlight the most promising phytochemical compounds belonging to two major classes, polyphenols and monoterpenes, and to report the main findings about their mechanisms of action relating to the AD pathogenesis.

## Introduction

Alzheimer’s disease (AD) is one of the most common age-related diseases. It currently affects over 40 million people worldwide, accounting for up to 70% of all cases of dementia (Sengupta et al., 2016; Bhatti et al., 2019; Zhang et al., 2021). The AD syndrome is characterized by the progressive deterioration of cognitive abilities accompanied by an irreversible neuronal loss (Selkoe and Hardy, 2016). The AD clinical symptomathology, described for the first time by Alois Alzheimer in 1907 (Alzheimer, 1907), includes memory impairment, behavioral alterations, language deficits, and other symptoms (Querfurth and LaFerla, 2010). AD represents a devastating brain disorder with a significant burden for health systems. Unfortunately, the few drugs approved for the AD treatment are only limited to a slight relief of symptoms but are not able to arrest the onset and progression of the AD-related neurodegeneration (Husna Ibrahim et al., 2020; Jeremic et al., 2021). For these reasons, much attention is still being given to drug development and to a deeper understanding of the AD pathophysiological processes. Many efforts are currently made by researchers from around the world to find new molecular targets for the development of effective therapies. However, one of the major limitations of any treatment is the fact that at the time of the clinical diagnosis an irreversible brain atrophy has already occurred and the cascade of events leading to neurodegeneration is well developed (Perrin et al., 2009; Holtzman et al., 2011).

The formation of AD lesions develops over two or 3 decades, resulting in a preclinical period in which the AD neuropathology is present without any cognitive symptoms. At the moment of the clinical diagnosis, the brain of AD patients is mainly characterized by large extracellular deposits of aggregated Amyloid-β (Aβ) peptides, also called amyloid plaques, and by degenerating neurons containing neurofibrillary tangles (NFTs), which are mainly composed of the microtubule-associated protein Tau in a hyperphosphorylated form (Selkoe and Hardy, 2016; Trejo-Lopez et al., 2021). Beside these two principal hallmarks, additional changes may be observed, including amyloid deposition at the vascular level (amyloid angiopathy), neuroinflammation, and a significant shrinkage of the hippocampus and enthorinal cortex, detectable at the MRI scan, in comparison to the brain of unaffected people (Serrano-Pozo et al., 2014).

The knowledge about the etiology of AD remains still incomplete. Three genes whose mutations induce an aberrant processing of the amyloid precursor protein (APP) and the consequent increase of the Aβ production, the APP, Presenilin 1 (PS1) and Presenilin 2 (PS2) genes, have been identified as the main determinants of the early-onset autosomal dominant AD (Serretti et al., 2007; Fernandez et al., 2014). Regarding the late-onset forms of familial AD, non-dominant genetic factors have been found to account for most of the cases (Kwok et al., 2020). By contrast, still little is known about the origins of the Aβ deposition and neurodegeneration occurring in the sporadic AD, which represents the greatest proportion of all AD cases (Efthymiou and Goate, 2017). Nonetheless, the inheritance of the ε4 allele of the Apolipoprotein (APO) E gene, which is associated with both increased Aβ deposition and tau pathology (Shi et al., 2017; Shi et al., 2021), constitutes the strongest genetic risk factor for sporadic AD, determining the age at onset of the disease and strictly influencing its progression (Cerf et al., 2011; Hashimoto et al., 2012; Koffie et al., 2012). The increasing age is instead considered the major non-genetic risk factor, with genetic and environmental factors influencing the onset and severity of the disease (Zhang et al., 2021).

At present, the most accepted etiological hypothesis of AD is the so-called “amyloid cascade hypothesis,” which considers the aggregation of the Aβ peptide and its accumulation in brain tissues as the main trigger of the AD neurodegenerative cascade (Selkoe and Hardy, 2016). According to this hypothesis, the intracellular and extracellular deposition of Aβ aggregates triggers a sequence of deleterious events that contribute to the AD pathogenesis, including gliosis, oxidative stress, Tau hyperphosphorylation, neuronal death, and synaptic loss (Braak and Braak, 1994). However, the “amyloid cascade hypothesis” has progressively undergone an extensive debate because of its inability to fully describe the complex pathogenesis of AD and the continuous failure of Aβ-targeted therapies. Moreover, it has been shown that the Tau pathology could cause neurodegeneration and neuroinflammation regardless of the Aβ pathology (Kametani and Hasegawa, 2018; Laurent et al., 2018). Meanwhile, several other hypotheses have been proposed, including those centered on the impairment of cholinergic transmission, mitochondrial dysfunction, oxidative stress, neuroinflammation, and brain insulin resistance. However, the common limitation of these hypotheses is that, similarly to the “amyloid cascade hypothesis,” they overemphasize a single specific mechanism, underestimating other mechanisms. In this regard, it is worth noting that much information about the pathological features of AD arises from the numerous genetic and toxin-induced rodent models of AD that have been developed in the last decades. These models, despite replicating some AD symptoms and being useful for testing the efficacy of anti-AD drugs, are not sufficient for modeling the complexity of the AD pathology and, importantly, are incapable to explain the origins of the sporadic forms of AD.

**FIGURE 1:**
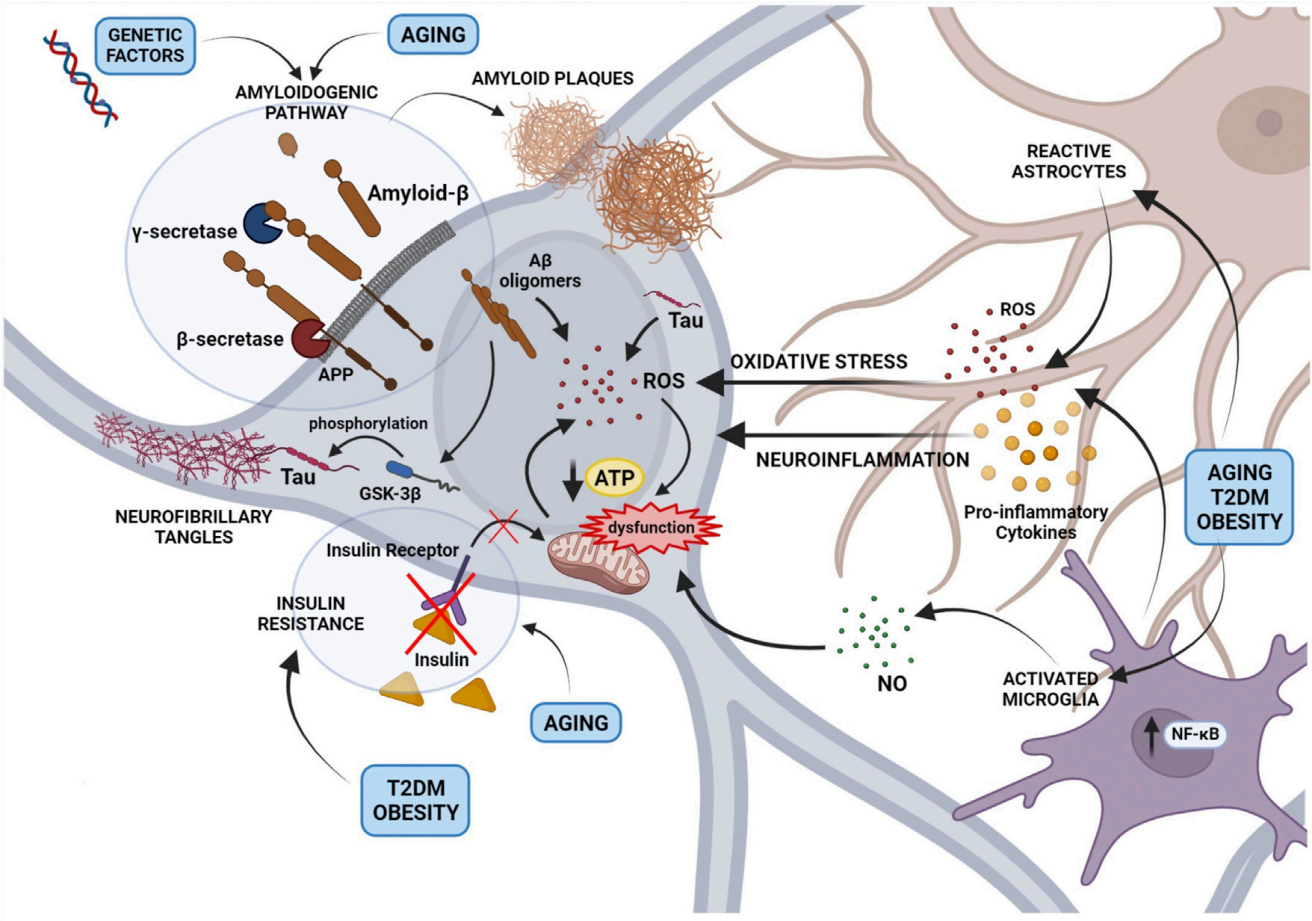
An overview of the main mechanisms involved in Alzheimer’s disease pathogenesis and their interplay according to a multi-factorial hypothesis. The figure depicts the role of the amyloidogenic processing of the Amyloid Precursor Protein (APP) induced by genetic factors or aging, in the formation of Amyloid-β (Aβ) oligomers and extracellular amyloid plaques. Small Aβ aggregates contribute to reactive oxygen species (ROS) production and mitochondrial dysfunction, formation of Tau aggregates and neurofibrillary tangles (NFTs). In addition, the activation of astrocytes and microglia, resulting in the release of cytokines, ROS, and nitric oxide, contribute to neuronal oxidative stress and mitochondrial dysfunction. Mitochondrial damage caused by a neuroinflammatory milieau, aging or metabolic disorders such as Type 2 diabetes mellitus and obesity induces the accumulation of free radicals and impairs the energetic efficiency of the neuron.

A “multifactorial hypothesis” of AD assumes that multiple etiological factors may converge in a common pathological manifestation (Figure 1). This seems to be the most convincing model able to explain why all the drugs developed under the “one-molecule, one-target” paradigm have displayed poor therapeutic potential. In addition, although how the complex interplay among different mechanisms contribute to the AD etiology remains still poorly understood, a multifactorial hypothesis is probably the most suitable to describe the onset of sporadic AD taking also note of aging as the most important risk factor. In this view, a therapeutic strategy targeting simultaneously different pathways considered as determinants of the AD pathogenesis seems to be the most promising approach to modify the disease progression. In this context, plant-derived compounds are emerging as an enormous source to draw on in the search for multi-target agents completing or assisting the traditional pharmacological medicine. The attempt of this review is therefore to highlight the most promising phytochemicals for the AD treatment belonging to two major classes, polyphenols and monoterpenes, and to describe their mechanisms of action.

## Main Pathogenic Mechanisms in AD

### Aβ Aggregation and Toxicity

The Aβ aggregation has been extensively investigated in the last 2 decades (Selkoe, 1991; Selkoe and Hardy, 2016) and most efforts to prevent or slow down the AD progression have been centered thus far on the inhibition of this process. The extracellular deposits of Aβ aggregates, namely the neuritic plaques, are the primary hallmarks found in the brain of the patients diagnosed with AD (Selkoe and Hardy, 2016). However, the Aβ aggregation and accumulation start at the cellular level many years before the clinical diagnosis of AD (Morris et al., 1996; Brannstrom et al., 2014).

The Aβ peptide, consisting of 39–43 amino acid residues, is produced in the brain upon the cleavage of the APP by proteolitic enzymes. The APP, which has been reported to be involved in many important physiological functions of neurons (Priller et al., 2006), is a large transmembrane protein physiologically undergoing a non-amyloidogenic processing consisting in the sequential cleavage by α- and γ-secretases (Selkoe, 2002). Importantly, the cleavage site for the α-secretase lies within the Aβ sequence of the APP and hence precludes the Aβ formation. In the so-called “amyloidogenic pathway” the APP is instead processed first by the β-secretase, also known as β-site APP Cleaving Enzyme 1 (BACE-1), and then by the γ-secretase, which generates monomeric Aβ fragments. The most abundant species are the Aβ_1-40_ and Aβ_1-42_ (Seubert et al., 1993), the latter being the most prone to aggregate into structures with a β-sheet conformation and, hence, the most toxic (Bressler et al., 1996; Folch et al., 2018). The total Aβ burden depends on the balance between production and clearance or degradation rates (Hardy, 1997; George-Hyslop and Rossor, 2001). The Aβ clearance may occur through the transport into the cerebrospinal fluid, the blood across the blood-brain barrier (BBB), and the removal by brain-resident macrophages (Yoon and Jo, 2012). The Aβ degradation is ensured by some proteases, such as cathepsin and the insulin-degrading enzyme (IDE), which cleave the Aβ into smaller soluble fragments (Miners et al., 2011; Kurochkin et al., 2018).

Both Aβ_1-40_ and Aβ_1-42_ have been shown to interact with several receptors including the N-Methyl-D-Aspartate (NMDA), α-Amino-3-hydroxy-5-Methyl-4-isoxazole Propionic acid (AMPA), nicotinic Acetylcholine (nACh), and muscarinic Acetylcholine (mACh) receptors (Tozaki et al., 2002; Snyder et al., 2005; Liskowsky and Schliebs, 2006; Gu et al., 2009), and to induce a synaptic transmission impairment through Ca^2+^ dysregulation and the blockage of ion channels and neurotransmitters (Peña et al., 2006; Pannaccione et al., 2020). The aggregation of Aβ_1-42_ monomers first gives rise to soluble oligomeric species, which further assemble to form insoluble aggregates known as fibrils accumulating in the brain parenchyma and other tissues (Chen et al., 2017). In an alternative aggregation pathway known as secondary nucleation, the fibrils act as a catalytic surface for the formation of new aggregates (Arosio et al., 2015). The toxicity of Aβ_1-42_ aggregates is directed to different cell types and affects a myriad of cellular functions. Oxidative stress, mitochondrial dysfunction, astrocytic and microglial activation, membrane perturbation, and loss of ionic homeostasis are, at the cellular level, some of the events downstream the Aβ_1-42_ aggregation and accumulation (Selkoe and Hardy, 2016). All these processes converge in the disruption of neuronal network, synaptic dysfunction, neuroinflammation, and neurodegeneration, leading in turn to the loss of cognitive abilities and, ultimately, in the severe dementia typical of the late stages of AD (Palop et al., 2007; Busche et al., 2015; Zott et al., 2018; Li and Selkoe, 2020; de Oliveira et al., 2021).

According to the so-called “cellular phase,” an extension of the amyloid cascade hypothesis proposed by De Strooper and Karran (De Strooper and Karran, 2016), the dysfunction of the neuro-glial-vascular unit along with the impairment of astrocytic and microglial homeostatic functions mainly contribute to precipitate AD in a clinical disease. Importantly, although the neuro-glial-vascular dysfunction and subsequent cerebrovascular damage are also believed to precede and even contribute to the disruption of Aβ homeostasis, the soluble Aβ aggregates spreading throughout the brain may in fact form insoluble deposits at the vascular level and compromise the vascular function and BBB integrity (Xu et al., 2016; Soto-Rojas, et al., 2021). Unsurprisingly, such a condition, known as cerebral amyloid angiopathy exacerbates the AD pathology (Shabir et al., 2018). Glial cells seem to play a key role in this process, since both astrocytes, the predominant form of glial cells in the central nervous system (CNS), and microglia, the major phagocytic cells in the brain, are involved in the tight regulation of Aβ clearance and degradation. Importantly, the capability of astrocytes and microglia to internalize and degrade Aβ (Guénette, 2003; Hansen et al., 2018) is strictly correlated with their aberrant activation during the AD progression (Bates et al., 2009; Ries and Sastre, 2016). Moreover, Aβ has been recognized to exert oxidative stress in astrocytes by altering astrocytic Ca^2+^ homeostasis and mitochondrial function (Abramov et al., 2004; Verkhratsky et al., 2017; Piccialli et al., 2020). In turn, the Aβ-induced dysregulation of ionic homeostasis in astroglial cells may crucially contribute to their pathological remodelling (Boscia et al., 2017; Verkhratsky et al., 2017; Piccialli et al., 2020).

### Tau Hyperphosphorylation and Toxicity

Multiple lines of evidence suggest that the hyperphosphorylation and aggregation of the protein Tau may be a major trigger of AD neurodegeneration. Tau, the main component of NFTs, is a microtubule stabilizer protein that normally undergoes a myriad of post-translational modifications, including phosphorylation (Goodson and Jonasson, 2018). Different kinases have been demonstrated to be involved in Tau phosphorylation, including the glycogen synthase kinase 3β (GSK-3β), protein kinase A, p38 mitogen activated protein kinase (MAPK), extracellular signal-related kinases (Erk1/2), and c-Jun N-terminal kinases (JNK) 1/3 (Martin et al., 2013). In AD, an imbalance between kinase and phosphatase activities causes Tau hyperphosphorylation. It is known that Aβ aggregates may induce Tau hyperphosphorylation by enhancing the activity of several kinases including GSK-3β and MAPKs. Moreover, Aβ triggers the activation of caspase-3 and calpain-1, which can cleave Tau at the C-terminus generating small fragments able to induce neurite degeneration and neuronal death (Gamblin et al., 2003; Bloom, 2014; Chen et al., 2018). Interestingly, the activation of the JNK pathway induces caspase activation, further promoting Tau cleavage (Sahara et al., 2008). Tau hyperphosphorylation leads to its aggregation and dissociation from microtubules (Wang et al., 2013; Gao et al., 2018). The dissociation from microtubules and the formation of NFTs in turn cause the impairment of the axonal transport, cytoskeletal and mitochondrial dysfunction, oxidative stress, and synaptic loss (Hoover et al., 2010) and, moreover, increase the cytosolic content of tau further promoting its aggregation and fibrillization. Similarly to Aβ, aggregated Tau forms β-sheet-containing amyloid fibrils, known as paired helical filaments (Mandelkow et al., 2007). Another noteworthy feature of Tau is its ability to spread throughout the brain by moving from 1 cell to another, anatomically connected, cell hence perfectly exemplifying the prion-like propagation characteristic of several proteinopathies (Dujardin and Hyman, 2019). Importantly, Tau can affect mitochondrial function by inducing ROS production, reducing mitochondrial respiration and causing the impairment of mitochondrial transport along neuronal axons (David et al., 2005; Dubey et al., 2008). Although Tau toxicity is considered a major driver of AD neurodegenerative cascade, there is still a significant gap in the knowledge of the molecular mechanimsms underlying tau-mediated neuroinflammation. Nonetheless, very recently it has been demonstrated that Tau induces a pro-inflammatory response in microglia by activating p38 MAPK, whose blockade, instead, promotes microglial phagocytic function and reverses Tau toxicity (Perea et al., 2018; Perea et al., 2022).

### Oxidative Stress and Mitochondrial Dysfunction

Oxidative stress and mitochondrial dysfunction are prominent features of many neurodegenerative diseases including AD (Butterfield, 2002; Butterfield and Boyd-Kimball, 2018; Sharma and Kim, 2021). Reactive oxygen species (ROS) and reactive nitrogen species (RNS), which exist in both radical and non-radical forms, are very reactive molecules that may influence cell functions and viability depending on their concentration. Mitochondria are widely recognized as a major source of ROS, since reactive species are physiologically produced through mitochondrial respiration. In particular, the leak of protons from some complexes of the mitochondrial electron transport chain and their interaction with oxygen (O_2_), normally occurring during the process of energy production, provide the majority of ROS in the cell. Physiologically, ROS and RNS concentration is maintained at low levels by enzymatic and non-enzymatic factors. Among the enzymes, the superoxide dismutase (SOD) plays a crucial role in antioxidant processes by converting the superoxide radical O_2_•−, a very reactive specie, to H_2_O_2_, a more stable form of ROS. Glutathione peroxidase (GPx) and catalases, in turn, limit the accumulation of H_2_O_2_ by producing water and oxygen. Glutathione (GSH), which acts as a cofactor of GPx, is also a non-enzymatic antioxidant able to quickly inactivate reactive species by itself. At physiological concentrations, ROS play a key role as second messengers, by oxidizing the–SH group on the cysteine residues of proteins and hence regulating their post-translation modifications, activity, and trafficking. In particular, ROS have been associated to an increased activity of MAPK pathways, such as those involving Erk1/2, JNK, and p38 MAPK. Moreover, ROS may impact the activity of several transcription factors sensitive to redox changes including the Hypoxia Inducible Factor-1α (HIF-1α) and the nuclear factor erythroid 2-related factor 2 (Nrf2).

The brain is particularly susceptible to oxidative stress because of the high content of lipids, although the different brain cell types do not display the same vulnerability to oxidative damage. Glial cells are indeed more resistant to oxidative injury, while neurons, especially those in the amygdala and hippocampus, are more sensitive. In neuronal cells, a rise in intracellular ROS levels causes protein degradation, DNA damage, and lipid peroxidation. In this context, mitochondria are a major target of ROS/RNS damages (Hung et al., 2018). Indeed, in addition to induce protein carbonylation, lipid peroxidation, and mitochondrial DNA damage (Marchi et al., 2012), ROS and RNS directly trigger the opening of the permeability transition pore (PTP), which in turn amplifies free radical signals. In addition, the exposure to reactive species can result in a decrease in the enzymatic activity of the complexes of the respiratory chain, thus reducing the efficacy of the production of ATP and further increasing the generation of ROS (Zorov et al., 2014). The occurrence of a severe mitochondrial dysfunction in the brain of AD patients has been extensively demonstrated (Abate et al., 2020). In particular, high levels of sporadic mutations in mitochondrial DNA were found in AD brains along with deficiencies in mitochondrial DNA repair mechanisms (Lin et al., 2002; Singh et al., 2015; Nissanka and Moraes, 2018). Alongside, many components of the antioxidant system have been shown to be particularly affected during the AD progression. At molecular level, both oxidative stress and mitochondrial failure have been linked to Aβ, although it is still unclear whether the Aβ aggregation and accumulation are predominantly the cause or rather a consequence of oxidative stress (Cheignon et al., 2018). Undoubtedly, Aβ oligomers may induce ROS production through several mechanisms. First, the Aβ peptide is oxidative *per se* because of the peculiar chemical/electrostatic features of its secondary structure. For the same reason, the Aβ insertion into the lipid bilayer of the neuronal plasma membrane induces lipid peroxidation in a direct manner (Butterfield, 2002). Furthermore, Aβ oligomers induce an increase of intracellular Ca^2+^ levels promoting its accumulation in mitochondria (Calvo-Rodriguez et al., 2019), which in turn induces the opening of the mPTP, release of mitochondrial ROS, and apoptotic cell death (Esteras and Abramov, 2020).

On the other hand, many experimental data suggest that oxidative stress and mitochondrial dysfunction induce an increase in the amyloidogenic cleavage of the APP and an aberrant Aβ production (Misonou et al., 2000; Perez Ortiz and Swerdlow, 2019). Indeed, it was shown that oxidizing agents can increase the expression of the APP (McCarty et al., 2021), and the expression and activity of BACE-1 (Tamagno et al., 2002; Tong et al., 2005; Zhang et al., 2016). Moreover, both *in vitro* and *in vivo* studies pointed out the Aβ peptide as a target molecule of metal-catalyzed oxidation (Näslund et al., 1994; Kowalik-Jankowska et al., 2004; Inoue et al., 2009). However, whether Aβ oxidation impacts on its aggregation and/or its binding to plasma membranes remains to be elucidated.

### Neuroinflammation

Neuroinflammation is a physiological process aimed at protecting the CNS from several injuries and involves several types of cells and mediators. A growing amount of evidence has revealed that a sustained inflammatory state in the brain is a major contributor in the AD pathogenesis (Dhapola et al., 2021). Furthermore, neuroinflammation is a well-documented downstream event of the Aβ accumulation (Akiyama et al., 2000). Accordingly, several studies reported high levels of inflammatory markers in the brain of AD patients, elevated levels of cytokines and chemokines in serum and cerebrospinal fluid, and microgliosis (Zhang F et al., 2013; Dursun et al., 2015; Hesse et al., 2016). Importantly, the increase of these markers seems to be correlated with the cognitive impairment occurring in AD (Solfrizzi et al., 2006; Westin et al., 2012).

The neuroinflammatory process is characterized by the activation of glial cells, especially astrocytes and microglia that, once activated, trigger the release of cytokines and chemokines such as interleukin-1β (IL-1β) and interleukin-6 (IL-6), and other pro-inflammatory mediators including the nuclear factor-κB (NF-κB), nitric oxide (NO), tumor necrosis factor (TNF)-α, TNF-β, adhesion molecules, and enzymes like cyclooxygenase-2 (COX-2) (Akiyama et al., 2000). The role of activated microglial cells is crucial in neuroinflammation since they can exert both neurotoxic and neuroprotective effects depending on the phenotype they acquire upon activation (Woodburn et al., 2021). Importantly, it has been demonstrated that soluble Aβ oligomers can activate microglia and the production of pro-inflammatory cytokines (Selkoe and Hardy, 2016; Yang et al., 2017). On the other hand, it has been reported that neuroinflammation and the subsequent impairment of the neuro-glial-vascular unit, which is mainly composed of neuronal, glial, and vascular cells supporting the BBB (Harder et al., 2002), occur before the disruption of the Aβ homeostasis (Zlokovic, 2011). Indeed, the BBB disruption and increased permeability have been shown to occur in the early phase of the AD pathology (van de Haar et al., 2016). The increased leakage from the BBB may impair the Aβ clearance and lead to microglial activation, hence amplifying the inflammatory response (Janota et al., 2016).

NF-κB nuclear transcription factor plays a crucial role in neuroinflammation, as it is the master regulator of the transcription of pro-inflammatory genes. Moreover, it has also been proposed as a molecular factor underlying some sporadic forms of AD (Chen et al., 2012). NF-κB is responsible for the activation of uncontrolled microglia, with subsequent ROS production, and pro-inflammatory cytokine and glutamate release, which in turn induce neuronal damage (Spencer et al., 2012). In particular, microglia stimulate the formation of NO by the inducible NO synthase (iNOS). NO, in turn, triggers neurodegenerative processes since it reduces the ATP synthesis by blocking the neuronal mitochondrial respiration at the cytochrome C oxidase level, hence inducing the production of ROS. Another crucial role is played by those signaling pathways involving MAPKs. The p38 MAPK, which is activated by the ROS produced upon microglial activation, was shown to activate NF-κB (Kheiri et al., 2018). Importantly, it has been reported that the Aβ-induced oxidative stress leads to the activation of p38 MAPK and subsequent tau hyperphosphorylation (Giraldo et al., 2014).

In addition, decreased levels of Nrf2, a transcription factor negatively regulated by NF-κB (Liu et al., 2008) that is involved in antioxidants response mechanisms (Nair et al., 2008), have been reported in the brains of AD patients (Ramsey et al., 2007). Based on the evidence that an Nrf2 deficiency increases the levels of pro-inflammatory mediators, there is a growing interest in finding any Nrf2 pharmacological activators.

### Brain Insulin Resistance

Many age-related brain abnormalities at molecular and cellular levels have been identified as contributing factors of sporadic AD. Among those attracting particular attention, the impairment of brain glucose/energy metabolism induced by metabolic disorders such as obesity, diabetes and hyperinsulinemia, has been widely accepted to be implicated in the AD progression (de la Monte, 2017; Ettcheto et al., 2020). The deterioration of systemic glucose metabolism, which may range from chronic, mild glucose intolerance to type 2 diabetes mellitus (T2DM), has been found to be present before the clinical appearance of sporadic AD, hence suggesting that an early and severe brain hypometabolism may contribute to the neuropathological changes leading to AD (Cunnane et al., 2011). Despite its ability to uptake glucose in an insulin-independent manner, the brain is in fact an insulin-sensitive organ widely expressing insulin receptors (IR), especially in those regions considered crucial for cognition and feeding (Milstein and Ferris, 2021). Accordingly, it is now clear that both insulin and its analogue, the insulin-like growth factor 1 (IGF-1), can influence brain energy homeostasis, neuronal survival, and learning and memory processes (Kapogiannis and Mattson, 2011). For this reason, insulin resistance, which consists in a lower insulin activity at the cellular level accompanied by an impaired metabolism of carbohydrates, lipids, and proteins (DeFronzo et al., 2015), can have a crucial impact on cognitive functions. High fat diet (HFD) feeding, obesity and T2DM are the main causes of insulin resistance and are all recognized as risk factors for late-onset AD (Terzo et al., 2021). T2DM is a complex chronic disorder highly prominent in older people, characterized by the chronic increase in blood glucose levels, impaired insulin secretion by pancreatic β-cells, and insulin resistance (DeFronzo et al., 2015). T2DM is also associated to hyperlipidaemia, resulting from the use of lipids instead of glucose, and to subsequent microvascular and macrovascular complications (Vesa et al., 2020). Importantly, epidemiological studies indicate that T2DM is associated to an increased risk to develop AD in comparison to normal people (Biessels et al., 2006).

Obesity is a multifactorial disorder of energy metabolism often associated with other pathological conditions including dyslipidemia, hypertension, and stroke (Hall et al., 2015). Obesity may induce brain insulin resistance through several mechanisms: 1) in obesity conditions the adipose tissue loses its ability to store fatty acids, thereby inducing their accumulation in other tissues including the brain, which consequently develop insulin resistance (Engin, 2017); 2) in the adipose tissue of obese people, macrophages become pro-inflammatory and produce inflammatory cytokines, which crossing the BBB induce central inflammation and subsequent brain insulin resistance (De Souza et al., 2005; Zatterale et al., 2020); 3) nutrients overload induces a mitochondrial fatty acids and glucose accumulation leading to mitochondrial dysfunction, subsequent ROS production, and oxidative stress, which in turn contribute to the reduction of insulin signaling and inflammation (Marìn-Royo et al., 2019).

Insulin resistance has also been found in association with AD independently on coincident T2DM (Bosco et al., 2011). In the brain of AD patients, IRs and IGF-1 receptors were found to be significantly reduced along with a decrease of many components of the insulin signaling cascade (Steen et al., 2005). This evidence led to consider AD as a brain diabetes or a “Type 3 diabetes”. Although there is evidence supporting some roles of insulin in regulating the brain glucose uptake, it is well known that the brain preferentially expresses the insulin-insensitive glucose transporters 1 and 3, while the insulin-sensitive glucose transporter 4 (GLUT4) is limited to specific brain regions such as the hippocampus and hypothalamus (Leloup et al., 1996; Reno et al., 2017). However, insulin signaling crucially affects many aspects of mitochondrial metabolism, which in turn plays a central role in controlling cellular metabolism and nutrient sensing. For this reason, brain insulin resistance induces the impairment of mitochondrial metabolism and, subsequently, the reduction of the ATP production, mitochondrial dyshomeostasis and increased ROS production (Sripetchwandee et al., 2018). It is also worth noting that insulin resistance leads to a reduced Aβ degradation by IDE, hence promoting the Aβ accumulation (Ho et al., 2004; Zhao et al., 2004; Petrov et al., 2015). In addition, the impairment of insulin signaling induces an increased activation of GSK-3β, which phosphorylates Tau, hence leading to Tau hyperphosphorylation (Hong and Lee, 1997; Planel et al., 2007; Zhang et al., 2018; Wei et al., 2021).

## The Search For Disease-Modifying Strategies in Alzheimer’s Disease: Multi-Target Therapies And Life-Style Interventions

**FIGURE 2:**
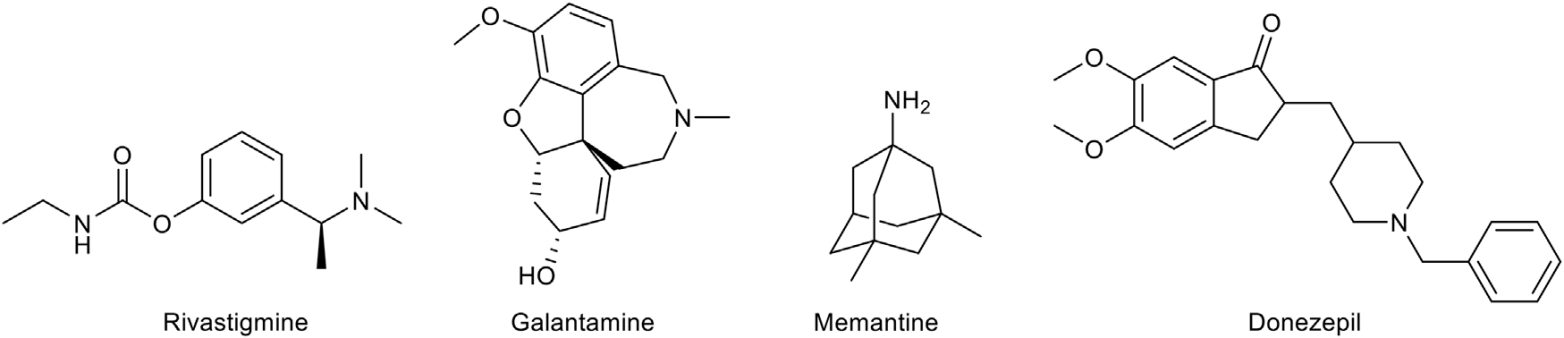
Chemical structures of the approved drugs for Alzheimer’s disease treatment.

As mentioned above, there is a limited number of drugs for the treatment of AD. The pharmacological agents currently used for the therapy of the AD-associated dementia comprise three acetylcholinesterase (AChE) inhibitors, donezepil, rivastigmine, and galantamine (Figure 2). The only non-AChE inhibitor drug, memantine, is a NMDA receptor antagonist (Figure 2). All these drugs are used in clinical practice for mild to severe dementia and provide a symptomatic relief alleviating to some extent the cognitive symptomathology. However, despite useful in improving the quality of life of the patients, they are not able to interrupt or delay the AD progression. This evidence has prompted the search for effective disease-modifying agents.

To date, the majority of the disease-modifying strategies for AD have been based on Aβ-focused approaches aimed at interfering with the Aβ production, deposition and clearance. In particular, some drugs targeting the Aβ peptide have been developed to control the APP proteolitic cleavage by β- and γ-secretases. Different BACE-1 inhibitors entered clinical trials because of the excellent results in preclinical studies. However, these trials proved unsuccessful due to the lack of effectiveness, as for the BACE-1 inhibitor Lanabecestat (Miranda et al., 2021), or because of a cognitive worsening in the patients, as in the case of Umibecestat (Neumann et al., 2018). Similarly, γ-secretase inhibitors failed clinical trials because of many side effects (Miranda et al., 2021). As alternative approaches, the inhibition of the Aβ aggregation and the destabilization of pre-existing Aβ aggregates have also been considered. Two drugs able to inhibit the Aβ aggregation, tramiprosate and colostrinin, showed promising results in the early phases of clinical trials to fail successively in displaying any efficacy (Bilikiewicz and Gaus, 2004; Aisen et al., 2007).

Beside the anti-aggregating molecules, other therapeutic strategies have included active or passive immunization against the Aβ peptide, consisting in the administration of Aβ antigens and anti-Aβ monoclonal antibodies, respectively. Unfortunately, although most of the Aβ-targeted drugs tested in clinical trials unveiled promising results in reducing the Aβ burden, they did not prove a significant efficacy against the cognitive decline, hence raising the question of whether targeting solely the Aβ peptide could really impact on the AD pathogenesis. Since Tau neurofibrillary tangles are the other key pathological hallmark of AD, many pharmacological strategies have been directed to the Tau pathology, by addressing Tau hyperphosphorylation through Tau kinase inhibitors such as GSK-3β inhibitors, or its aggregation by means of anti-aggregating molecules (Long and Holtzman, 2019). Indeed, although it was postulated that the altered Aβ metabolism precedes the Tau-related pathology and neuronal degeneration, many reports showed that Tau hyperphosphorylation is a primary factor in AD independently on Aβ, and that the formation of amyloid plaques and neurofibrillary tangles may in fact act in parallel or in combination to contribute to the development of AD (Bloom, 2014). On the other hand, since Tau neurofibrillary tangles and amyloid plaques are not unique to AD but are also characteristic of the aging brain as well as of other diseases, it has been proposed that both these hallmarks may be pathological manifestations of other underlying mechanisms causing the disease.

According to the assumption that aging constitutes a predominant risk factor for AD, many studies have highlighted that increased levels of oxidative stress, mitochondrial dysfunction, neuroinflammation, and metabolic alterations, which are prominent features of aging, may all be critical in the initiation of the neurodegeneration occurring in AD (Song et al., 2021). In addition, a broad range of comorbidities and the underlying biological mechanisms have been shown to affect the AD pathogenesis and progression, including obesity, diabetes, cardiovascular diseases, stroke, and depression (Santiago and Potashkin, 2021). In the light of this evidence, the conventional approach based on the use of single-target drugs is currently switching toward a multi-target strategy able to interfere with different aspects of the AD pathogenesis (Oset-Gasque and Marco-Contelles, 2018). From one side, the multi-target approach may be achieved through a combination therapy, in which a cocktail of multiple single-target agents is administered. Such strategy, however, retains some limitations, including the higher risk of drug-drug interactions, which in turn may affect the metabolism of the administered drugs and, hence, alter their efficacy and/or toxicity. From the other side, the pharmacological research is paying growing attention on the development of pleiotropic ligands interacting with two or more therapeutic targets at the same time. To this aim, a new drug design strategy is being considered, which consists in the combination of different pharmacophores to obtain hybrid molecules with the ability to modulate different pathways.

A great emphasis has also been placed on non-pharmacological interventions able to prevent or reduce AD severity. Many observational studies on AD animals and human subjects evidenced that life-style interventions including physical exercise, caloric restriction and nutritional supplements with antioxidant compounds are effective in reducing the modifiable AD risk factors, such as those raising from aging processes or any comorbidities (Bhatti et al., 2019; Dominguez et al., 2021). Novel strategies conceived to prevent the AD onset are focusing on dietary changes and nutritional supplements (Dominguez et al., 2021). In particular, there is a growing body of evidence supporting the ability of dietary polyphenols, such as those found in the Mediterranean diet, to interfere with different AD-related pathomechanisms and to slow down age-related cognitive deficits hence reducing the risk of dementia (Casamenti and Stefani, 2017; Bagetta et al., 2020). Epidemiological studies indicate that the consumption of flavonoids, a large family of polyphenol compounds, may ameliorate the AD pathology and provide a symptomatic benefit especially due to their anti-inflammatory and antioxidant activities (Devi et al., 2021; Hole and Williams, 2021).

## Plant-Derived Natural Compounds as Multi-Target Disease-Modifying Agents

Natural products have played a dominant role over the centuries in the search for new therapeutic agents for the treatment of a wide range of human diseases. Natural compounds may indeed help in the discovery of new lead compounds or serve as structural mimics for synthetic analogues. Recently, a great emphasis on testing natural medicine for the AD therapy has been developed. Of note, galantamine, a tertiary alkaloid first isolated from *Galanthus nivalis* L. (Amaryllidaceae), is currently included in the AD therapy for its cholinesterase inhibitory properties and its ability to enhance the cholinergic function and ameliorate memory deficits (Heinrich, 2010; Anand and Singh, 2013). Rivastigmine, instead, is a semi-synthetic drug approved by the FDA as a cholinesterase inhibitor recommended for mild-to-moderate AD (Marucci et al., 2021). Based on this promising evidence and on the growing need for natural alternatives with less adverse effects in comparison to pharmaceutical drugs, phytochemical compounds are gaining attention as possible components of preventive or combination therapies aimed at counteracting the AD neurodegeneration (Jurcau, 2021; Lye et al., 2021). Many findings have already been provided by the literature about the efficacy of several natural compounds including polyphenols and monoterpenes in affecting different pathogenic mechanisms of AD thanks to their antioxidant and anti-inflammatory properties, and to the ability to modulate the Aβ aggregation and toxicity. Many of them have also been demonstrated to act as multi-target agents with reported fewer side effects in comparison to their synthetic counterparts (Rahman et al., 2021).

A huge number of phenolic compounds have displayed the capability to interfere with the aggregation of the Aβ peptide by targeting one or multiple steps (Ma et al., 2020; Stefanescu et al., 2020). In particular, the action of natural inhibitors can be directed to 1) the assembly of Aβ monomers, producing small aggregates; 2) the remodeling of Aβ oligomers, leading to the formation of off-pathway species; 3) the inhibition of the secondary nucleation (Najarzadeh et al., 2019). These actions are underlain by the direct interaction of the compounds with the Aβ peptide through covalent or non-covalent bindings (Henriquez et al., 2020; Ma et al., 2020). In particular, the phenolic moieties can establish π-π stacking with the aromatic residue of the Aβ or establish hydrophobic or hydrogen bonding interactions through phenolic hydroxyl groups (Fan et al., 2020). It was also demonstrated that the higher the number of catechol moieties the more the inhibitory activity increases (Tsunoda et al., 2018). Biophysical and docking studies also demonstrated that a guaiacol moiety is required for the anti-aggregating activity of polyphenolic inhibitors (Tomaselli et al., 2019). As mentioned above, the presence of anti-inflammatory and antioxidant properties is another crucial feature of natural compounds contributing to their therapeutic potential (Uddin et al., 2021). Many phytochemicals including flavonoids and other polyphenols, terpenoids, and carotenoids have indeed the ability to target multiple signaling pathways responsible for microglial activation and the subsequent release of pro-inflammatory mediators, or those involved in the activation of NF-κB and p38 MAPK (Spilsbury et al., 2012; Spagnuolo et al., 2018; Jin et al., 2019; Olajide and Sarker, 2020). Other natural products are able to activate Nrf2, a mechanism that has been shown to underlie at least in part their anti-inflammatory activity. Overwhelming evidence has also highlighted the use of natural compounds with a strong antioxidant activity as a promising approach for the AD treatment (Guan et al., 2021; Walia et al., 2021). However, evidence of their efficacy has been quite limited thus far possibly due to a poor bioavailability or to the time point in which the treatment is undertaken (Forman and Zhang, 2021). For these reasons, many debates have developed on how the bioavailability of these compounds may be improved and whether a dietary supplementation with natural antioxidant could be more useful than their employment for a therapeutical intervention (Fusco et al., 2007; Feng and Wang, 2012; Denzer et al., 2016; Pallauf et al., 2017). Among phytochemicals, flavonoids are largely known for their antioxidant activity as they can act directly as reactive oxygen scavengers by donating hydrogen. Nevertheless, flavonoids display a low circulating concentration if compared to well-characterized ROS scavengers, hence suggesting that their antioxidant potential could be due to different mechanisms such as the modulation of intracellular pathways involved in oxidative stress (Reiber et al., 1993; Borges et al., 2018). Importantly, many natural substances, especially polyphenols, have been shown to be effective in counteracting the main pathophysiological mechanisms of T2DM hence offering potential benefits for reducing the risk of both diabetes and AD (Blahova et al., 2021). These substances may represent a complementary approach, along with life-style intervention such as exercise and nutrition, aimed at enhancing glucose control, lowering insulin resistance and improving insulin sensitivity.

### Polyphenols

**FIGURE 3:**
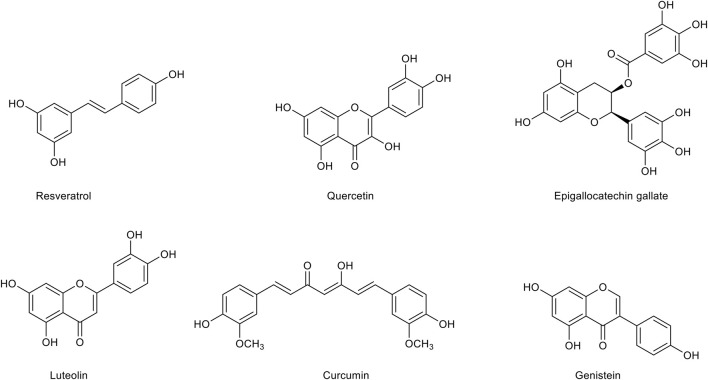
Chemical structures of the main polyphenolic compounds with a therapeutic potential for Alzheimer’s disease treatment.

Polyphenols (Figure 3) are a heterogeneous group of chemical substances naturally synthesized by the secondary metabolism of plants characterized by one or more aromatic rings bearing one or more hydroxyl groups. Flavonoids, the most diffuse phenolic compounds, are attracting growing attention for their multiple benefits. Indeed, many studies have correlated flavonoid intake with the improvement of cognition, attenuation of neuroinflammation and oxidative stress (Cichon et al., 2021). Although there is still uncertainty about their pharmacokinetics properties, flavonoids and other polyphenols are currently investigated as possible therapeutic agents in the AD treatment. Indeed, while on one hand many studies demonstrated that nano-incapsulation of flavonoids may overcome pharmacokinetic limitations and improve their bioavailability, on the other hand these compounds are also considered as attractive molecular scaffolds for the development of more potent and specific therapeutics.

Resveratrol, a polyphenol typically found in the skin of grapes (Vitis vinifera L., Vitaceae), has gained increasing interest for its therapeutic potential in metabolic diseases and for its ameliorative effect on cognitive decline. Indeed, resveratrol displays different pharmacological effects, including anti-inflammatory, antioxidant and anti-diabetic effects. Resveratrol has been shown to exert an inhibitory activity on neuroinflammation in several studies (Candelario-Jalil et al., 2007; Lu et al., 2010; Potter et al., 2013; Zhang R et al., 2013; Wang et al., 2015). In particular, it has been shown to inhibit the production of pro-inflammatory factors such as NO, TNF-α and IL-1β in both astrocytic and microglial cells (Yao et al., 2015; Zhao et al., 2018). *In vivo* studies have demonstrated that resveratrol is able to improve cognition and reduce amyloid plaque formation in Tg6799 mice (Chen et al., 2019), to inhibit microglial activation in APP/PS1 mice (Capiralla et al., 2012), and to decrease the amount of insoluble Aβ in the hippocampus of AD rats, with positive effects on BBB integrity (Zhao et al., 2015). Moreover, resveratrol has been demonstrated to affect the Aβ production by inhibiting the activity of β-secretase (Choi et al., 2011; Koukoulitsa et al., 2016) and to modulate the Aβ clearance through several mechanisms (Zhang et al., 2014). Further studies have shown that resveratrol is able to inhibit the Aβ aggregation from lower molecular weight (MW) oligomers into higher MW oligomers and to disrupt pre-formed Aβ aggregates (Fu et al., 2014; Ghobeh et al., 2014). The beneficial effects of resveratrol in metabolic disorders such as insulin resistance and T2DM have also been characterized. Data from the literature indicate that resveratrol ingestion is able to stimulate glucose uptake by increasing the translocation of GLUT4 (Szkudelski and Szkudelska, 2011). Moreover, resveratrol has been suggested to counteract the HFD-induced insulin resistance (Mendez-del Villar et al., 2014). Interestingly, the treatment with resveratrol in streptozotocin (STZ)-injected diabetic rats has been able to counteract many pathogenic mechanisms, including oxidative stress, inflammation and synaptic loss, and to prevent memory impairment by modulating cholinergic transmission (Tian Z. et al., 2016; Tian et al., 2016 Y.). In a clinical trial, the treatment with resveratrol of patients with mild-to-moderate AD induced a decrease of Aβ levels in cerebrospinal fluid and a reduction of neuroinflammation biomarkers (Moussa et al., 2017). However, a pilot study carried on elderly subjects has revealed that the chronic use of resveratrol selectively improves psychomotor speed without significantly ameliorate memory function (Anton et al., 2018). Nonetheless, this result could be connected to the poor penetration of the BBB and an overall low oral bioavailability. Therefore, formulations of resveratrol with an improved bioavailability could increase its efficacy. In particular, the nano-incapsulation in lipid carriers or liposomes as well as the insertion into polymeric particles could ameliorate both pharmacodynamics and pharmacokinetics of resveratrol (Andrade et al., 2018). Moreover, some efforts have been made to synthesize various analogues of resveratrol with a more potent biological activity (Lee et al., 2012; Lu et al., 2013; Biscussi et al., 2020).

Quercetin, a ubiquitous flavonoid widely distributed in fruits and vegetables, has been found to exert strong anti-aggregating and anti-inflammatory activities. Quercetin has been proven to effectively inhibit the Aβ aggregation by destabilizing the oligomeric species of misfolded proteins and inhibiting the fibril growth (Matsuzaki et al., 2007). Quercetin is also able to reduce iNOS-mediated NO production in lipopolysaccharide (LPS)-treated BV2 microglial cells by suppressing NF-κB activation (Kang et al., 2013). Moreover, quercetin has been demonstrated to attenuate the inflammation mediated by IL-1β in astrocytes and neuronal cultures by inhibiting the release of IL-6 and IL-8 (Sharma et al., 2007). The anti-inflammatory activity of quercetin has also been thought to underlie its positive effect on cognitive function in APP/PS1 mouse model of AD, where it also reduces Aβ plaques and Tau phosphorylation (Lv et al., 2018). In 3xTg-AD mice, quercetin decreases the amount of the extracellular Aβ, improves astrogliosis and microgliosis, and preserves cognitive function (Sabogal-Guáqueta et al., 2015; Barreca et al., 2016). The putative antioxidant potential of quercetin has also been considered for its use in AD treatment since it has both a direct antioxidant activity, due to two pharmacophores present in the molecule able to scavenge free radicals, and the ability to modulate antioxidant pathways. Indeed, quercetin has been shown to act as a scavenger of both ROS and RNS (Boots et al., 2008), to increase the expression of SOD and GSH (Ishige et al., 2001), and to positively modulate Nrf2 signaling (Arredondo et al., 2010; Molaei et al., 2020; Yu et al., 2020). Both *in vitro* and *in vivo* studies have shown that quercetin exerts beneficial effects on diabetes. In particular, it has been shown that quercetin reduces blood glucose levels, increases plasma insulin levels and positively affects memory and learning functions in streptozocin (STZ)-induced diabetic rats (Khaki et al., 2010; Srinivasan et al., 2018; Yang and Kang, 2018). Unfortunately, despite its promising therapeutic potential, a clinical approach with the use of quercetin has been quite limited probably due to its poor permeability of the BBB, low bioavailability, and rapid metabolism (Cai et al., 2013). However, several studies using multiple nanoparticles formulations have shown that the use of nanotechnology may significantly improve the brain delivery of quercetin and enhance its bioavailability (Kumari et al., 2010; Bagad and Khan, 2015; Najafabadi et al., 2018).

Luteolin, a flavone widely distributed in herbs and vegetables, has emerged as an interesting phytochemical able to mitigate many pathogenic mechanisms responsible for AD, including neuroinflammatory processes and the impairment of brain glucose metabolism. Luteolin seems to be a modulator of immune reactions for its very efficient anti-inflammatory properties in peripheral macrophages, compared to those of other flavonoids including its analogue quercetin (Comalada et al., 2006). Interestingly, luteolin exerts an anti-inflammatory effect also in LPS-activated microglia where it has been found to significantly reduce the expression of iNOS and COX-2 and to downregulate pro-inflammatory cytokines and the production of NO (Zhu et al., 2011). This finding has been confirmed in *in vivo* studies. In particular, it has been shown that a dietary administration of luteolin reduces microglial activation in senescent mice (Burton et al., 2016). This effect has been explained by the ability of luteolin to suppress in a dose-dependent manner the expression of pro-inflammatory and pro-apoptotic genes hence shifting the microglial transcriptome toward an anti-inflammatory phenotype (Dirscherl et al., 2010). Recently, it has been reported that luteolin is able to improve memory deficits in a mouse model of AD by inhibiting astrocyte overactivation and neuroinflammation and by reducing endoplasmic reticulum (ER) stress (Kou et al., 2021). In agreement, luteolin has been demonstrated to improve cognitive abilities in a mouse model of vascular dementia by modulating the expression of pro-inflammatory and pro-apoptotic proteins and by stimulating neurogenesis (Siracusa et al., 2017; Cordaro et al., 2020). Very interesting, it has been shown that the combination of luteolin with another anti-inflammatory compound, the palmitoylethanolamide is able to reduce iNOS and GFAP expression and protect glial cells against the Aβ injury in both *in vitro* and *ex vivo* experimental models (Paterniti et al., 2014). The effective anti-inflammatory activity of luteolin has also been suggested to contribute to its protective effect against insulin resistance (Daily et al., 2021). Indeed, luteolin has been shown to improve insulin sensitivity in many cell types, including adipocytes and endothelial cells (Ding et al., 2010; Deqiu et al., 2011). In agreement, a diet supplemented with luteolin has been demonstrated to ameliorate obesity and insulin resistance in mice (Xu et al., 2014). In obese mice, luteolin improves cognitive deficits by alleviating neuroinflammation, oxidative stress and neuronal insulin resistance (Liu et al., 2014). Interestingly, the combination of luteolin with the L-theanine amino acid prevents AD-like symptoms by improving the hippocampal insulin signaling and decreasing neuroinflammation in Aβ-infused rats (Park et al., 2018).

Epigallocatechin gallate (EGCG), the main polyphenolic constituent of the tea plant *Camelia sinensis* (L.) Kuntze (Theaceae), is considered the major responsible for the beneficial effects of green tea. Many *in vitro* and *in vivo* studies have demonstrated that EGCG and its metabolites may exert significant neuroprotective activities. Several experimental data have highlighted the ability of EGCG to interfere with the Aβ aggregation. EGCG can bind weakly and in a non-specific manner to Aβ monomers while it displays higher affinity toward oligomers (Ahmed et al., 2017). Electron microscopy studies have shown that ECGC can inhibit the Aβ secondary nucleation and intervene in the early steps of the Aβ aggregation inducing the formation of spherical, off-pathway aggregates. (Ehrnhoefer et al., 2008; Ahmed et al., 2017). It is still unclear, instead, whether EGCG is able to disassemble preformed fibrils. On the other hand, it has been demonstrated that ECGC may disrupt Aβ protofibrils by forming π-π and hydrogen bonding interactions (Zhan et al., 2020; Li M et al., 2021). In transgenic APP_SWE_ Tg2576 mice, the intraperitoneal treatment with EGCG for 2 months is associated to a significant reduction of the Aβ levels, although it has been suggested that this result arises from the ability of EGCG to modulate the APP cleavage pathway (Rezai-Zadeh et al., 2005). In particular, experimental studies have provided evidence that EGCG increases the α-cleavage of APP (Rezai-Zadeh et al., 2005; Ettcheto et al., 2020). EGCG has also displayed an anti-inflammatory activity in several studies by reducing TNF-α, IL-1β, IL-6, and iNOS levels and rescuing neurogenesis through mechanisms involving NF-κB (Cheng-Chung Wei et al., 2016; Seong et al., 2016). It has also been demonstrated that EGCG exerts a positive effect on cognitive function by promoting ROS scavenging (Schroeder et al., 2009) and counteracting the Aβ-induced mitochondrial apoptotic processes (Fukutomi et al., 2021). Importantly, it has recently been reported that EGCG treatment ameliorates mitochondrial respiration deficits in children affected by Down syndrome (Scala et al., 2021). A recent meta-analysis of 17 studies performed on animal models of AD has demonstrated that EGCG can rescue cognitive impairment and Aβ pathology through the combination of its anti-inflammatory, antioxidant, and antiaggregating activities (Zhang et al., 2020). EGCG has been also shown to act as an anti-diabetic agent especially due to its significant anti-inflammatory and antioxidant activities. In particular, EGCG is able to increase glucose tolerance and the glucose-stimulated insulin secretion in obese mice (Ortsäter et al., 2012) and to affect diabetes-related comorbidities such as diabetic neuropathy (Raposo et al., 2015). Interestingly, EGCG treatment improves insulin sensitivity and memory deficits in APP/PS1 mice fed with a HFD (Ettcheto et al., 2020). Unfortunately, clinical results on the efficacy of EGCG in ameliorating cognitive functions have not reflected the therapeutic potential emerged in pre-clinical studies. In fact, EGCG treatment displays some limitations such as the high rate of metabolism and subsequent degradation, which in turn prevent a sufficient concentration in human plasma (Pervin et al., 2019). On the other hand, EGCG displays to permeate the BBB at low micromolar levels (Pervin et al., 2017).

Curcumin, a polyphenol extracted from the rhizomes of *Curcuma longa* L. (Zingiberaceae), possesses a variety of pharmacological properties. Growing evidence has demonstrated that curcumin can significantly improve cognitive functions in AD by reducing oxidative damage, inflammation and Aβ aggregation. In particular, it has been reported that curcumin inhibits the formation of Aβ_1-40_ and Aβ_1-42_ fibrils *in vitro* in a dose-dependent manner and destabilizes preformed fibrils (Ono et al., 2004; Yang et al., 2005; Hamaguchi et al., 2010). Different studies have shown that curcumin is able to bind monomeric species and low MW oligomers (Fu et al., 2014) and to induce major structural changes in the Aβ_1-42_ aggregates (Mithu et al., 2014). More recently, it has been demonstrated that curcumin and its derivatives are able to intercalate among Aβ chains in the first stage of the Aβ aggregation and to form hydrogen bonding and hydrophobic interactions with the Aβ peptide, leading to more disordered amyloid structures (Doytchinova et al., 2020). Interestingly, *in vivo* studies have shown that the systemic treatment with curcumin reduces pre-existing plaques in ∼8-month-old APPswe/PS1dE9 mice, suggesting its ability to disaggregate Aβ deposits (Garcia-Alloza et al., 2007). Curcumin effects have also been investigated in *in vitro* and *in vivo* models of neuroinflammation. In particular, curcumin has been demonstrated to inhibit NF-κB and MAPK activation in LPS-stimulated microglia (Porro et al., 2019; Zhang et al., 2019). These findings have been confirmed by the results obtained in an animal model of AD showing that the treatment with curcumin ameliorates neuroinflammation and cognitive decline upon exposure to LPS (Sorrenti et al., 2018). In an *in vitro* study, curcumin has been shown to decrease in a concentration-dependent manner the expression of IL-1β, IL-6 and TNF-α in the Aβ-activated microglia by inhibiting Erk1/2 and p38 MAPK pathways (Shi et al., 2015). Curcumin is also capable to control oxidative stress. In particular, the antioxidant activity of curcumin seems to derive from its β-diketone structure and phenolic groups, which confer the ability to scavenge free radicals (Ak and Gülçin, 2008). In neuronal cultures exposed to pro-oxidant conditions, curcumin exerts a strong neuroprotective effect by counteracting the toxicity of H_2_O_2_ and Fe^3+^ (Morales et al., 2017). In rat pheochromotocytoma cells (PC12) exposed to Aβ toxicity, curcumin has been shown to inhibit oxidative damage (Park et al., 2008). The therapeutic potential of curcumin has been further confirmed by several studies on animal models of insulin resistance. Indeed, curcumin has been demonstrated to increase the activity of insulin receptors in rats and activate insulin pathways (Li et al., 2010), and to delay T2DM by improving β-cell function and reducing insulin resistance (Chuengsamarn et al., 2012). Interestingly, the use of curcumin as such in clinical studies does not display a significant efficacy against AD, although the drug is well-tolerated (Ringman et al., 2012). This evidence has been explained with its poor ability to pass the BBB and its very low oral bioavailability (Begum et al., 2008). Therefore, in the last years many curcumin analogues with improved aqueous solubility, pharmacokinetics, bioavailability and stability have been synthesized. Importantly, these compounds show greater efficacy than curcumin hence appearing as promising agents for the AD treatment (Anand et al., 2008; Airoldi et al., 2011; Yanagisawa et al., 2011). Meanwhile, strategies to enhance curcumin delivery have also been evaluated. In particular, curcumin-containing nanoliposomes as well as other delivery systems have been designed and show to be very effective in reducing the Aβ fibrils formation (Taylor et al., 2011; Lazar et al., 2013; Mourtas et al., 2014; Shahbaz et al., 2021).

Genistein is a naturally occurring isoflavone mainly found in soybean, green peas, legumes, and peanuts. Mechanistically, genistein potentially inhibits the activity of tyrosine protein kinases and of a DNA topoisomerases. Additionally, owing to the presence of numerous phenolic moieties in its structure, genistein exerts potent anti-oxidant effects (Gong et al., 2015). Therefore, genistein can modulate several pathogenetic mechanisms in AD including Aβ metabolism, inflammation and cholinergic system dysfunction. Accordingly, genistein can reduce the production of Aβ by inhibiting BACE-1 (Youn et al., 2018). Furthermore, Hirohata et al. (2012) observed that genistein can directly bind the Aβ_25-35_ fragment preventing the formation of its aggregates. Genistein can reduce the neurotoxicity induced by Aβ_1-42_ by inhibiting kinesin AP180T (AP180) and Ras homolog family member A (RhoA) and, therefore, Aβ accumulation (Yu et al., 2010).

Neuroinflammation is responsible for an abnormal secretion of pro-inflammatory cytokines that trigger detrimental signaling pathways leading to protein aggregation in the AD brain. In this context, Valles et al. (2010) demonstrated that the increased levels of IL-1β and TNF-α induced by Aβ were modulated treating astrocytes with genistein (Valles et al., 2010). In addition, genistein can inhibit the activity of NF-kB signal pathway mediated by Toll-like receptor (TLR4) (Zhou et al., 2014). Accordingly, genistein blocks the binding of NF-kB to the DNA thus antagonizing the neuroinflammatory effect in AD (Seo et al., 2018). Interestingly, in diabetic mice genistein (Pedersen et al., 1996; Wang et al., 2016), as well as some of its derivatives (Qiang et al., 2014), can improve cognitive dysfunction by inhibiting the activity of AChE and by the modulation of Ca^2+^ homeostasis in cholinergic neurons (Jhamandas et al., 2001).

### Monoterpenes

**FIGURE 4:**
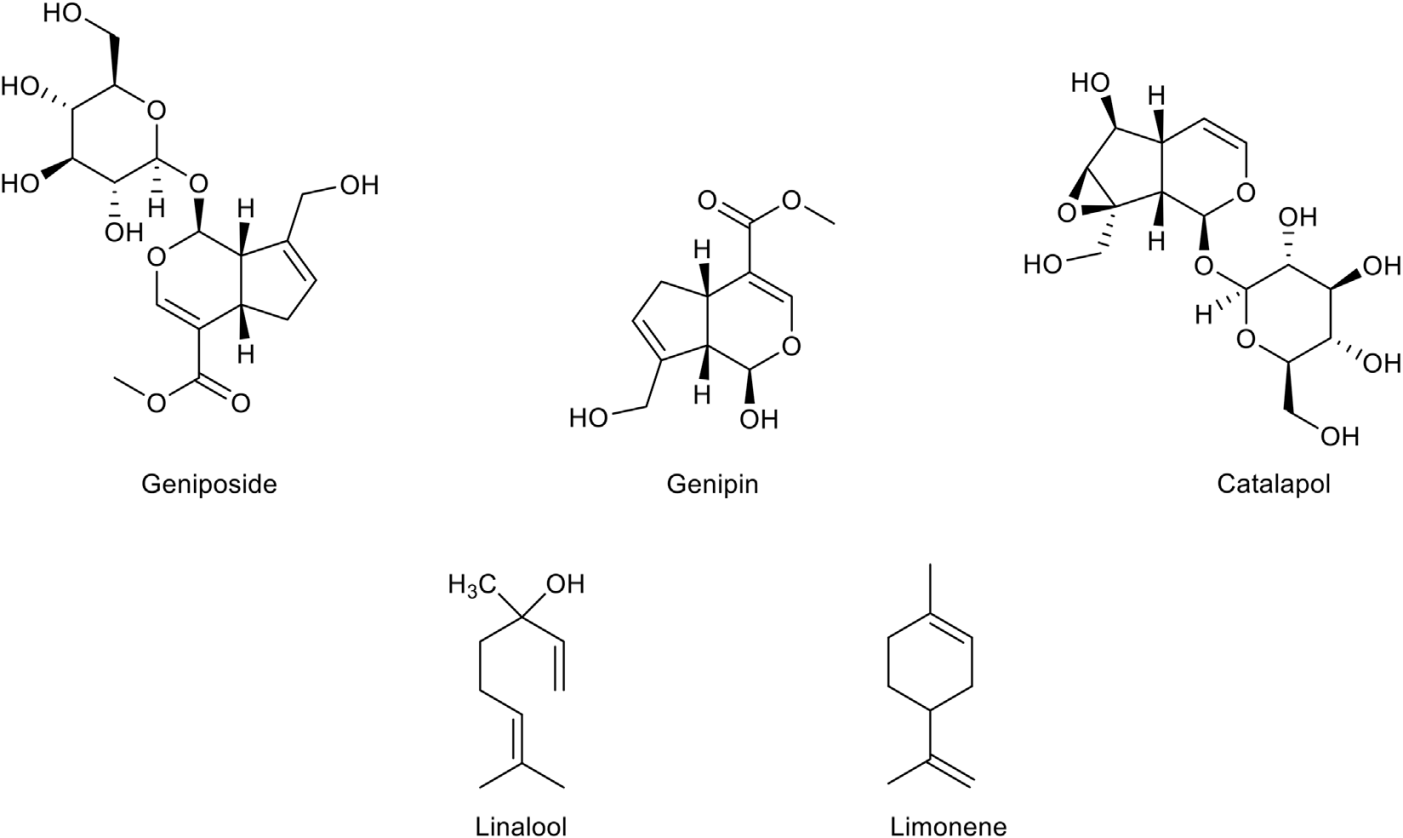
Chemical structure of the main monoterpenes with a therapeutic potential for Alzheimer’s disease treatment.

Monoterpenes (Figure 4 **)**, a group of isoprene derivatives also known as isoprenoids, are found as secondary metabolites in many aromatic plants. These plant-derived compounds, already known for their very effective antibacterial and antifungal activities, are now emerging as potential therapeutic agents in multiple disorders for their antioxidant activity. Many monoterpenes display a free radical scavenging ability, which seems to arise from the presence of the conjugated double bonds typical of the isoprenoid structure. In addition, the growing knowledge about supplementary activities of monoterpenes such as anti-inflammatory, anti-cholinesterase, and anti-nociceptive activities, is highlighting their therapeutic potential in several neurodegenerative diseases including AD.

Geniposide, a major iridoid glycoside of *Gardenia jasminoides* J. Ellis (Rubiaceae) fruits, displays a significant neuroprotective potential. First, geniposide exerts both antioxidant and anti-apoptotic effects. In particular, geniposide has been demonstrated to protect PC12 and SH-SY5Y cells from oxidative damage and apoptotic processes, by modulating the MAPK signaling pathways and caspases expression and by increasing the levels of SOD and GPx (Liu et al., 2006; Liu et al., 2007; Yin et al., 2010a; Yin et al., 2010b; Sun et al., 2013). Interestingly, geniposide protects primary hippocampal neurons from the Aβ1-42 toxicity by improving mitochondrial axonal trafficking and function and ameliorating synaptic damage (Li et al., 2014; Sun et al., 2014; Zhao et al., 2016; Zhang et al., 2017). Increasing studies have emphasized the ability of geniposide to act as an agonist of the GLP-1 receptor (GLP-1R) and to trigger different neuroprotective mechanisms upon GLP-1R activation, including the induction of insulin secretion, the increase of intracellular cyclic Adenosine Monophosphate (cAMP) levels and the activation of IDE gene expression (Liu et al., 2006; Liu et al., 2009; Liu et al., 2012; Yin et al., 2012; Li et al., 2018). Based on these results, geniposide is attracting growing interest among researchers. Therefore, a substantial number of studies have been performed in mouse models of AD and amyloidosis, significantly reinforcing the therapeutic potential of this substance in counteracting the neurodegenerative processes of AD. In STZ-induced diabetic rats the intragastric administration of geniposide has been demonstrated to improve insulin resistance and blood glucose levels, to increase the expression of IDE and to decrease Aβ1-42 levels (Liu et al., 2013). In agreement, the intracerebroventricular (ICV) injection of geniposide in STZ-treated rats was able to improve learning and memory deficits, attenuate Tau hyperphosphorylation and prevent subcellular and synaptic abnormalities by reducing the activity of GSK-3 enzyme (Gao et al., 2014). In agreement, further results have shown that geniposide counteracts the increase of Tau phosphorylation induced by the insulin deficiency in AD mice treated with STZ, by promoting GSK-3β phosphorylation and subsequent inactivation (Zhang et al., 2015). In APP/PS1 transgenic mice, the treatment with geniposide ameliorates memory deficits by significantly suppressing oxidative damage, mitochondrial function, and MAPK signaling overactivation (Lv et al., 2014; Zhao et al., 2017). Other studies performed in APP/PS1 have suggested that the inhibitory effect of geniposide on Tau hyperphosphorylation and Aβ1-42 production is due to an increase of leptin signaling (Liu et al., 2015; Liu et al., 2017). Very interestingly, the aglycone of geniposide, genipin, which can be produced through the metabolism of geniposide by intestinal microflora enzymes, has recently been reported to possess several pharmacological properties, such as anti-inflammatory, anti-diabetic and anti-depressive effects (Li et al., 2016). Genipin has been found to exert a strong anti-inflammatory activity in several models of inflammation by targeting iNOS and NF-κB. In particular, it has been demonstrated that genipin inhibits the increase of NO production and iNOS, COX-2, IL-1β and IL-6 expression in LPS-activated BV2 microglial cells (Araki et al., 2014). Besides, genipin has also been shown to inhibit inflammation by downregulating chemokines, chemokine receptors and interferon-induced protein expression (Li et al., 2012). Genipin has also displayed the ability to protect neuronal cells against the cytotoxicity of Aβ, H2O2 and rotenone by preventing widespread damage induced by the increase of ROS and RNS production (Yamazaki et al., 2001; Hughes et al., 2014). Moreover, it has recently been reported that genipin reduces Tau phosphorylation and fibrillation in Tau-overexpressing cells and decreases the Aβ generation in neuronal cells overexpressing the Swedish mutant APP, by inhibiting BACE-1 expression (Li F et al., 2021).

Catalpol, an iridoid glycoside extracted from the roots of *Rehmannia glutinosa* (Gaertn.) DC. (Plantaginaceae), has been shown to interfere with different mechanisms underlying many pathological conditions such as diabetes, atherosclerosis, and ischemic injury (Cai et al., 2016; Liu et al., 2016; Xue et al., 2016; Yan et al., 2018; Bai et al., 2019; Chen et al., 2020; He et al., 2021). Many reports have demonstrated that catalpol is protective against the oxidative stress and mitochondrial dysfunction induced by several neurotoxins including rotenone, MPP(+) and D-galactose, by increasing the activity of antioxidant factors, preventing the complex I activity reduction, and counteracting the lipid peroxidation (Mao et al., 2007; Tian et al., 2007; Zhang et al., 2007). Several *in vitro* studies have also shown that catalpol is able to protect astrocytes from H_2_O_2_ damage and to suppress neuroinflammation by inhibiting NF-κB activation and reducing iNOS activity in LPS-treated astrocytes and in mice with acute systemic inflammation induced by LPS (Bi et al., 2008; Zhang et al., 2009; Bi et al., 2013). Catalpol has also been reported to protect neuronal and glial cells by Aβ_1-42_ toxicity by preventing astrocytic and microglial activation and the release of pro-inflammatory factors such as ROS, NO and TNF-α, and by attenuating the mitochondrial-dependent neuronal apoptosis (Jiang et al., 2008; Liang et al., 2009). A large number of pharmacological studies performed in different mouse models have confirmed the neuroprotective potential of catalpol. In particular, in mice intraperitoneally injected with D-galactose to reproduce senescence, catalpol is able to improve cholinergic function, reduce inflammatory cytokines levels, prevent mitochondrial dysfunction, and improve learning and memory deficits. Catalpol has also been found to promote the activity of endogenous antioxidant enzymes and decrease ROS and NOS production (Zhang et al., 2007; Zhang X. et al., 2008; Zhang X. L. et al., 2008; Zhang et al., 2010; Zhang X et al., 2013). In mice injected with D-galactose and Aβ, the treatment with catalpol is able to inhibit oxidative stress and to reduce Aβ levels by regulating the activity of SOD, GPx and by increasing the expression of IDE, which is involved in the Aβ degradation (Huang et al., 2016). In addition, it has been shown that catalpol is able to reduce the BBB damage and subsequent hyperpermeability induced by Aβ_1-42_ fibrils and to increase the Aβ clearance (Liu et al., 2018). Interestingly, in neuronal cells overexpressing the Swedish mutant APP, catalpol has been found to increase the expression of the α-secretase, through an Erk/cAMP-response element binding protein (CREB) pathway, hence promoting the non-amyloidogenic processing of the APP and reducing the generation of Aβ (Wang et al., 2018). The anti-inflammatory activity of catalpol has also been associated to its anti-diabetic potential. Indeed, catalpol exerts an anti-hyperglicemic effect in STZ-induced diabetic rats (Huang et al., 2010) and ameliorates insulin resistance induced by HFD by suppressing the NF-κB pathway (Zhou et al., 2015).

Linalool is a major component of essential oils from many aromatic plants. Despite its lower antioxidant activity compared to other monoterpenes due to the lack of conjugated double bonds, linalool has been shown to improve the antioxidant potential of many essential oils by acting in synergy with other components (Wojtunik-Kulesza et al., 2019). The neuroprotective effect of linalool has been suggested in several *in vitro* and *in vivo* experimental models of neurodegeneration. Indeed, linalool has been shown to counteract the increase of ROS production and mitochondrial Ca^2+^ levels, to preserve mitochondrial integrity, and to reduce lipid peroxidation in immortalized neuronal cells exposed to glutamate (Sabogal-Guáqueta et al., 2019). Moreover, linalool significantly counteracts cell death and COX-2 increase in organotypic hippocampal slices exposed to NMDA (Sabogal-Guáqueta et al., 2019). In line with these results, it has been demonstrated that linalool is able to interfere with the glutamatergic transmission, hence displaying an anticonvulsivant effect (Brum et al., 2001). Interestingly, the intraperitoneal administration of linalool counteracts the Aβ-induced hippocampal injury and ameliorates cognitive deficits in Aβ-treated mice. Specifically, linalool attenuates caspase activation, oxidative stress, and the reduction of Nrf2 expression induced by Aβ (Xu et al., 2017). In addition, the chronic administration of linalool has been reported to reduce β-amyloidosis, astrogliosis and microgliosis and to improve spatial and learning memory in triple transgenic AD mice (3xTg-AD). These neuroprotective effects correlate with reduced levels of p38 MAPK, NOS2, COX-2, and IL-1β (Sabogal-Guáqueta et al., 2016). In line with these reports, *in vitro* results from our laboratory have shown that linalool is able to counteract the mitochondrial dehydrogenase activity reduction, increase of intracellular levels of ROS, and caspase-3 activation in PC12 cells exposed to Aβ_1-42_ oligomers. In agreement, *Lavandula angustifolia* and *Coriandrum sativum* essential oils containing linalool as major component have also displayed antioxidant and anti-apoptotic effects, hence confirming the beneficial potential of linalool in synergistic combination with other components (Caputo et al., 2021).

Limonene, a monocyclic monoterpene abundantly found in the genus *Citrus* (Rutaceae), displays several biological properties including antioxidant, anti-inflammatory and anti-nociceptive activities (Roberto et al., 2010; d’Alessio et al., 2013; Piccinelli et al., 2017). Recently, limonene has been investigated by the scientific community for its pharmacological effects in various chronic diseases including AD, multiple sclerosis, and epilepsy (Eddin et al., 2021). Recent reports have shown that limonene may exert a beneficial effect in neurodegenerative diseases due to its antioxidant and anti-inflammatory activities. Like many other monoterpenes, limonene has been found to exert an anti-AchE activity (Szwajgier and Baranowska-Wójcik, 2019; Piccialli et al., 2021). A recent study performed in a *Drosophila* model of AD has shown that limonene is able to counteract the Aβ_1-42_-induced neurotoxicity by decreasing ROS levels and hence preventing Erk phosphorylation. Moreover, limonene induces a significant decrease in the number of activated glial cells and in the expression of NO, thus also proving to exert an anti-inflammatory effect (Shin et al., 2020). In addition, limonene has been demonstrated to suppress oxidative stress and inflammation by inhibiting COX-2, iNOS, and NF-kB in doxorubicin-treated rats (Rehman et al., 2014). Interestingly, limonene also displays an efficacy in preventing or alleviating metabolic disorders in HFD-fed mice (Jing et al., 2013). In STZ-induced diabetic rats it exerts an anti-hyperglicemic effect, increases the activity of antioxidant enzymes such as SOD and GSH, and reduces the levels of lipid peroxidation, hence displaying a therapeutic potential in preventing diabetes complications (Murali et al., 2013; Bacanli et al., 2017). Very recently, a study from our group has shown that limonene is able to counteract the reduction of mitochondrial dehydrogenase activity, increase of intracellular ROS levels and nuclear morphology alterations induced by Aβ_1-42_ oligomers in primary cortical neurons (Piccialli et al., 2021). In the same study, the antioxidant activity of limonene has been crucial in preventing the increase of outward transient potassium currents, which were shown to play a critical role in triggering caspase activation and apoptotic cell death (Pannaccione et al., 2007). Moreover, our results also confirmed that limonene is able to exert an anti-AchE activity almost comparable to that of galantamine (Piccialli et al., 2021), thus highlighting its multi-target therapeutic potential.

## Translating Phytochemicals Into Pharmaceuticals: Ongoing Challenges

The natural compounds described above are only a short collection of all the plant-derived natural compounds showing beneficial effects in AD. Likely, the amount of attracting molecules to be tested against neurodegeneration is expected to increase considering the growing interest in this research field. However, although they appear as an encouraging alternative route compared to many synthetic drugs failing to definitively cure AD, natural products have failed as well to display impressive efficacy in clinics. Indeed, while there are a massive number of mechanistic studies suggesting the therapeutic potential of natural compounds in AD and other neurodegenerative diseases, clinical results from human trials have been mostly insufficient or disappointing. The discrepancies between pre-clinical research and clinical results suggest that scientific literature may probably overestimate the potential benefits of natural compounds on cognitive impairment. Nevertheless, the lack of significant results may stem from complex reasons. The first critical hindrance to the clinical use of natural compounds is the difficulty in extrapolating the dose required in humans from that providing a biological effect in *in vitro* or *in vivo* studies. This problem, in turn, arises from the different responsiveness of cell cultures and animal models to the activity of certain agents, and the necessity, given a broad spectrum of targets and activities for a single compound, to decipher which target is mechanistically more valid to reach the desired therapeutic effect and, based on that target, to identify the adequate dose. Additional *in vitro* and *in vivo* studies should be performed to validate the use of natural compounds in combination with other agents or drugs, a strategy that might boost their efficacy and succeed in providing more relevant results. Another crucial issue is represented by the pharmacokinetic properties of natural compounds that determine their bioavailability, an aspect gaining complexity when it comes to evaluating the efficacy of dietary supplementation with polyphenols, in which the overall dietary intake may be critical. As mentioned above, the major pharmacokinetic limitations leading to clinically irrelevant results include the poor ability to cross the BBB and rapid metabolism. In this regard, transformation and absorption at the gut level deserve much attention, especially in the evaluation of the use of polyphenols in a dietary intervention, since the composition of gut microbiota may strongly influence and even drive their pharmacological effects. Moreover, dietary polyphenols have recently been demonstrated to have a clinically relevant impact on many essential functions of human body, including the regulation of immune system and gut microbiota (Grosso, 2018; van der Merwe, 2021)—an aspect that further complicates the validation of certain natural compounds as pharmaceuticals for AD therapies. Lastly, significant limitations affecting the overall therapeutic potential of phytochemicals are also related to the design of clinical trials. Indeed, the identification of the exact mechanism of action and, hence, of a therapeutic target is of crucial importance for an accurate selection of the participants, which in turn may strongly affect the success of a clinical trial. Therefore, although the ongoing strategies to overcome the aforementioned limitations, namely the synthesis of derivatives with improved pharmacokinetic profiles and nanotechnology, may help achieve the auspicious results further efforts with implemented multi-disciplinary approaches are needed to realize the pharmaceutical potential of natural compounds. Nonetheless, they remain an attractive opportunity to optimistically look to in the search for new effective AD treatments.

## Conclusion

**FIGURE 5:**
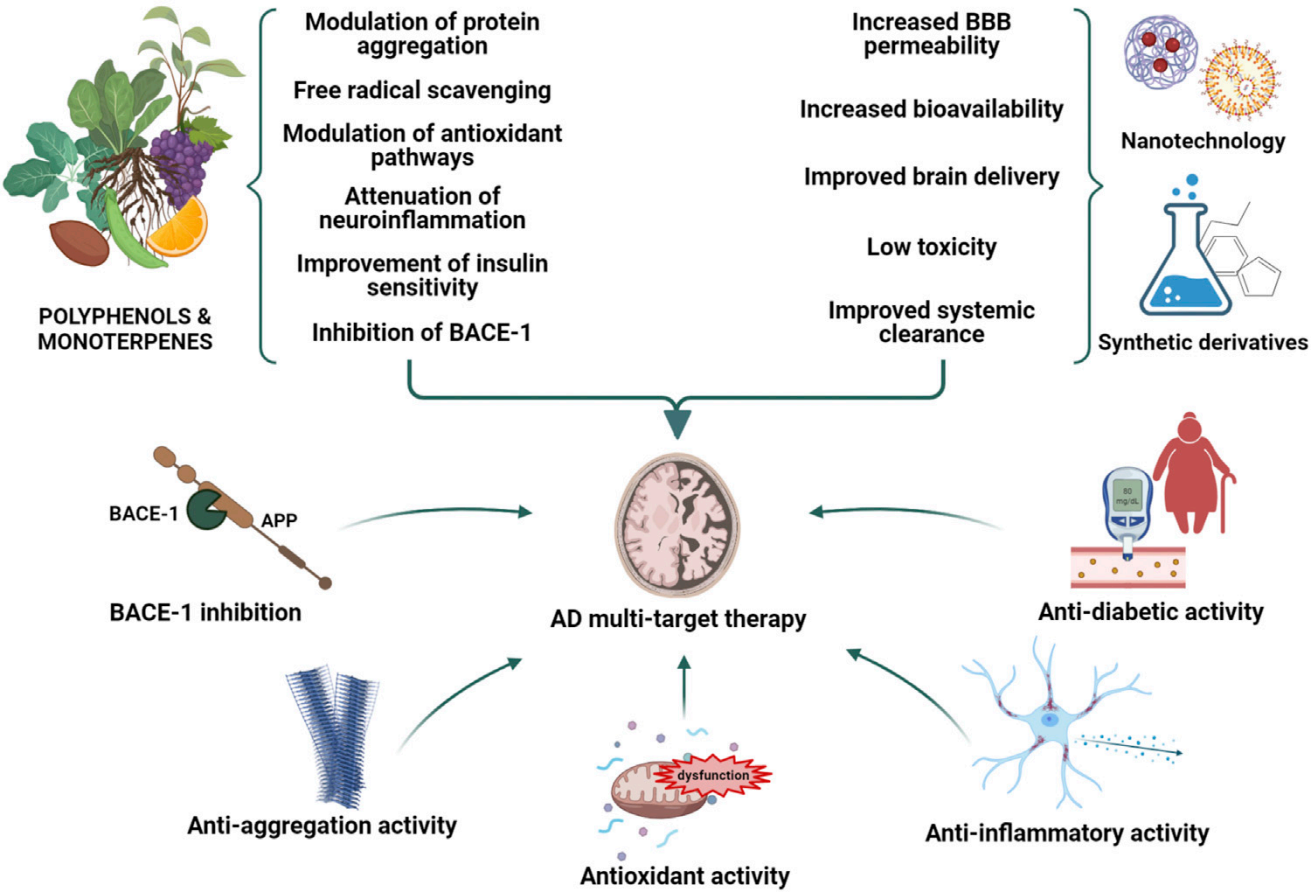
Polyphenols and monoterpenes as potential bioactive agents in Alzheimer’s disease (AD) multi-target therapy. Phytochemicals may serve as therapeutic agents to combat AD neurodegeneration owing to their antioxidant and anti-inflammatory activities, which may be useful in counteracting oxidative stress and neuroinflammation. In addition, they may modulate the Amyloid-β (Aβ) aggregation and accumulation hence reducing brain Aβ burden. The improvement of insulin-sensitivity by phytochemicals may also be beneficial for reversing the detrimental progression of AD. The combination of the biological activities of phytochemicals with nanotechnology and/or synthetic strategies may improve their pharmacokinetic profile and their therapeutic potential.

AD, along with many other age-related diseases, is the object of unceasing investigation due to its irreversibility and the lack of effective treatments. Considered the urgent need to find new neuroprotective strategies, many different routes have been approached. Natural products, which have been used for their medicinal properties since ancient times, have recently been researched for their ability to interfere with many biological processes and for their nutritional potential, both implicated in their therapeutic power. In the last decades several mechanistic and pilot studies have suggested the protective effect of natural products and their bioactive components against AD. In particular, many phytochemicals have been proven to exert significant antioxidant and anti-inflammatory effects. More recently, their ability to modulate the Aβ aggregation and to interfere with the amyloid cascade has also been demonstrated. Intriguingly, a major plus of many natural compounds is their effectiveness in regulating multiple molecular pathways, thus emerging as suitable agents for a multi-target therapeutic approach (Figure 5). To date, although preclinical investigations have suggested their neuroprotective potential, the clinical use of many promising plant-derived compounds has proven unsuccessful, especially due to their poor pharmacokinetic profile. To overcome this issue, both synthetic and nanotechnological strategies are currently taken into consideration. However, to translate pre-clinical results into clinics further issues should be addressed, including a better identification of the optimal dosage and formulation in order to obtain at the same time the strongest effect and the safest profile. A nutraceutical intervention with specific diets containing natural antioxidant and anti-inflammatory agents is also under investigation for its potential efficacy in preventing or reducing the risk of developing age-related neurodegenerative diseases. Nonetheless, the validation of a dietary supplementation with multi-active phytochemical compounds to treat or prevent AD also requires the examination of many critical issues such as those concerning the mutual influence between the dietary intake of such compounds and the regulation of many important functions of human body that may in turn affect the healthy state of the patients. Undoubtedly, the approval of a therapeutic approach centered on natural products is still in its infancy. Further efforts are needed to optimize the clinical relevance of phytochemicals and to realize their pharmaceutical potential.

## References

[B1] Abate G. Vezzoli M. Sandri M. Rungratanawanich W. Memo M. Uberti D. (2020). Mitochondria and Cellular Redox State on the Route from Ageing to Alzheimer's Disease. Mech. Ageing Dev. 192, 111385. 10.1016/j.mad.2020.111385 33129798

[B2] Abramov A. Y. Canevari L. Duchen M. R. (2004). Calcium Signals Induced by Amyloid Beta Peptide and Their Consequences in Neurons and Astrocytes in Culture. Biochim. Biophys. Acta 1742, 81–87. 10.1016/j.bbamcr.2004.09.006 15590058

[B3] Ahmed R. VanSchouwen B. Jafari N. Ni X. Ortega J. Melacini G. (2017). Molecular Mechanism for the (-)-Epigallocatechin Gallate-Induced Toxic to Nontoxic Remodeling of Aβ Oligomers. J. Am. Chem. Soc. 139, 13720–13734. 10.1021/jacs.7b05012 28841302

[B4] Airoldi C. Zona C. Sironi E. Colombo L. Messa M. Aurilia D. (2011). Curcumin Derivatives as New Ligands of Aβ Peptides. J. Biotechnol. 156, 317–324. 10.1016/j.jbiotec.2011.07.021 21807037

[B5] Aisen P. S. Gauthier S. Vellas B. Briand R. Saumier D. Laurin J. (2007). Alzhemed: a Potential Treatment for Alzheimer's Disease. Curr. Alzheimer Res. 4, 473–478. 10.2174/156720507781788882 17908052

[B6] Ak T. Gülçin I. (2008). Antioxidant and Radical Scavenging Properties of Curcumin. Chem. Biol. Interact. 174, 27–37. 10.1016/j.cbi.2008.05.003 18547552

[B7] Akiyama H. Barger S. Barnum S. Bradt B. Bauer J. Cole G. M. (2000). Inflammation and Alzheimer's Disease. Neurobiol. Aging 21, 383–421. 10.1016/s0197-4580(00)00124-x 10858586 PMC3887148

[B8] Alzheimer A. (1907). Über eine eigenartige Erkrankung derHirnrinde [On a peculiar, severe disease process of the cerebral cortex]. Allg. Z. Psychiat. 64, 146–148. English translation of excerpt given in Maurer’s biography on pages 160–162.

[B9] Anand P. Singh B. (2013). A Review on Cholinesterase Inhibitors for Alzheimer's Disease. Arch. Pharm. Res. 36, 375–399. 10.1007/s12272-013-0036-3 23435942

[B10] Anand P. Thomas S. G. Kunnumakkara A. B. Sundaram C. Harikumar K. B. Sung B. (2008). Biological Activities of Curcumin and its Analogues (Congeners) Made by Man and Mother Nature. Biochem. Pharmacol. 76, 1590–1611. 10.1016/j.bcp.2008.08.008 18775680

[B11] Andrade S. Ramalho M. J. Pereira M. D. C. Loureiro J. A. (2018). Resveratrol Brain Delivery for Neurological Disorders Prevention and Treatment. Front. Pharmacol. 9, 1261. 10.3389/fphar.2018.01261 30524273 PMC6262174

[B12] Anton S. D. Ebner N. Dzierzewski J. M. Zlatar Z. Z. Gurka M. J. Dotson V. M. (2018). Effects of 90 Days of Resveratrol Supplementation on Cognitive Function in Elders: A Pilot Study. J. Altern. Complement. Med. 24, 725–732. 10.1089/acm.2017.0398 29583015 PMC6065512

[B13] Araki R. Hiraki Y. Yabe T. (2014). Genipin Attenuates Lipopolysaccharide-Induced Persistent Changes of Emotional Behaviors and Neural Activation in the Hypothalamic Paraventricular Nucleus and the central Amygdala Nucleus. Eur. J. Pharmacol. 741, 1–7. 10.1016/j.ejphar.2014.07.038 25084220

[B14] Arosio P. Knowles T. P. Linse S. (2015). On the Lag Phase in Amyloid Fibril Formation. Phys. Chem. Chem. Phys. 17, 7606–7618. 10.1039/c4cp05563b 25719972 PMC4498454

[B15] Arredondo F. Echeverry C. Abin-Carriquiry J. A. Blasina F. Antúnez K. Jones D. P. (2010). After Cellular Internalization, Quercetin Causes Nrf2 Nuclear Translocation, Increases Glutathione Levels, and Prevents Neuronal Death against an Oxidative Insult. Free Radic. Biol. Med. 49, 738–747. 10.1016/j.freeradbiomed.2010.05.020 20554019

[B16] Bacanlı M. Anlar H. G. Aydın S. Çal T. Arı N. Ündeğer Bucurgat Ü. (2017). D-Limonene Ameliorates Diabetes and its Complications in Streptozotocin-Induced Diabetic Rats. Food Chem. Toxicol. 110, 434–442. 10.1016/j.fct.2017.09.020 28923438

[B17] Bagad M. Khan Z. A. (2015). Poly(n-butylcyanoacrylate) Nanoparticles for Oral Delivery of Quercetin: Preparation, Characterization, and Pharmacokinetics and Biodistribution Studies in Wistar Rats. Int. J. Nanomedicine 10, 3921–3935. 10.2147/IJN.S80706 26089668 PMC4468990

[B18] Bagetta D. Maruca A. Lupia A. Mesiti F. Catalano R. Romeo I. (2020). Mediterranean Products as Promising Source of Multi-Target Agents in the Treatment of Metabolic Syndrome. Eur. J. Med. Chem. 186, 111903. 10.1016/j.ejmech.2019.111903 31787360

[B19] Bai Y. Zhu R. Tian Y. Li R. Chen B. Zhang H. (2019). Catalpol in Diabetes and its Complications: A Review of Pharmacology, Pharmacokinetics, and Safety. Molecules 24, 3302. 10.3390/molecules24183302 PMC6767014 31514313

[B20] Barreca D. Bellocco E. D'Onofrio G. Nabavi S. F. Daglia M. Rastrelli L. (2016). Neuroprotective Effects of Quercetin: From Chemistry to Medicine. CNS Neurol. Disord. Drug Targets 15, 964–975. 10.2174/1871527315666160813175406 27528470

[B21] Bates K. A. Verdile G. Li Q. X. Ames D. Hudson P. Masters C. L. (2009). Clearance Mechanisms of Alzheimer's Amyloid-Beta Peptide: Implications for Therapeutic Design and Diagnostic Tests. Mol. Psychiatry 14, 469–486. 10.1038/mp.2008.96 18794889

[B23] Begum A. N. Jones M. R. Lim G. P. Morihara T. Kim P. Heath D. D. (2008). Curcumin Structure-Function, Bioavailability, and Efficacy in Models of Neuroinflammation and Alzheimer's Disease. J. Pharmacol. Exp. Ther. 326, 196–208. 10.1124/jpet.108.137455 18417733 PMC2527621

[B24] Bhatti G. K. Reddy A. P. Reddy P. H. Bhatti J. S. (2019). Lifestyle Modifications and Nutritional Interventions in Aging-Associated Cognitive Decline and Alzheimer's Disease. Front. Aging Neurosci. 11, 369. 10.3389/fnagi.2019.00369 31998117 PMC6966236

[B25] Bi J. Jiang B. Liu J. H. Lei C. Zhang X. L. An L. J. (2008). Protective Effects of Catalpol against H2O2-Induced Oxidative Stress in Astrocytes Primary Cultures. Neurosci. Lett. 442, 224–227. 10.1016/j.neulet.2008.07.029 18652878

[B26] Bi J. Jiang B. Zorn A. Zhao R. G. Liu P. An L. J. (2013). Catalpol Inhibits LPS Plus IFN-γ-Induced Inflammatory Response in Astrocytes Primary Cultures. Toxicol. Vitro 27, 543–550. 10.1016/j.tiv.2012.09.023 23164921

[B27] Biessels G. J. Staekenborg S. Brunner E. Brayne C. Scheltens P. (2006). Risk of Dementia in Diabetes Mellitus: a Systematic Review. Lancet Neurol. 5, 64–74. 10.1016/S1474-4422(05)70284-2 16361024

[B28] Bilikiewicz A. Gaus W. (2004). Colostrinin (A Naturally Occurring, Proline-Rich, Polypeptide Mixture) in the Treatment of Alzheimer's Disease. J. Alzheimers Dis. 6, 17–26. 10.3233/jad-2004-6103 15004324

[B29] Biscussi B. Richmond V. Baier C. J. Mañez P. A. Murray A. P. (2020). Design and Microwave-Assisted Synthesis of Aza-Resveratrol Analogues with Potent Cholinesterase Inhibition. CNS Neurol. Disord. Drug Targets 19, 630–641. 10.2174/1871527319666200905121536 32888280

[B30] Blahova J. Martiniakova M. Babikova M. Kovacova V. Mondockova V. Omelka R. (2021). Pharmaceutical Drugs and Natural Therapeutic Products for the Treatment of Type 2 Diabetes Mellitus. Pharmaceuticals (Basel) 14, 806. 10.3390/ph14080806 34451903 PMC8398612

[B31] Bloom G. S. (2014). Amyloid-β and Tau: the Trigger and Bullet in Alzheimer Disease Pathogenesis. JAMA Neurol. 71, 505–508. 10.1001/jamaneurol.2013.5847 24493463 PMC12908160

[B32] Boots A. W. Haenen G. R. Bast A. (2008). Health Effects of Quercetin: from Antioxidant to Nutraceutical. Eur. J. Pharmacol. 585, 325–337. 10.1016/j.ejphar.2008.03.008 18417116

[B33] Borges G. Ottaviani J. I. van der Hooft J. J. J. Schroeter H. Crozier A. (2018). Absorption, Metabolism, Distribution and Excretion of (-)-epicatechin: A Review of Recent Findings. Mol. Aspects Med. 61, 18–30. 10.1016/j.mam.2017.11.002 29126853

[B34] Boscia F. Pannaccione A. Ciccone R. Casamassa A. Franco C. Piccialli I. (2017). The Expression and Activity of KV3.4 Channel Subunits Are Precociously Upregulated in Astrocytes Exposed to Aβ Oligomers and in Astrocytes of Alzheimer's Disease Tg2576 Mice. Neurobiol. Aging 54, 187–198. 10.1016/j.neurobiolaging.2017.03.008 28390823

[B35] Bosco D. Fava A. Plastino M. Montalcini T. Pujia A. (2011). Possible Implications of Insulin Resistance and Glucose Metabolism in Alzheimer's Disease Pathogenesis. J. Cel. Mol. Med. 15, 1807–1821. 10.1111/j.1582-4934.2011.01318.x PMC3918038 21435176

[B36] Braak H. Braak E. (1994). Morphological Criteria for the Recognition of Alzheimer's Disease and the Distribution Pattern of Cortical Changes Related to This Disorder. Neurobiol. Aging 15, 355–380. discussion 379-380. 10.1016/0197-4580(94)90032-9 7936061

[B37] Brännström K. Öhman A. Nilsson L. Pihl M. Sandblad L. Olofsson A. (2014). The N-Terminal Region of Amyloid β Controls the Aggregation Rate and Fibril Stability at Low pH through a Gain of Function Mechanism. J. Am. Chem. Soc. 136, 10956–10964. 10.1021/ja503535m 25014209

[B38] Bressler S. L. Gray M. D. Sopher B. L. Hu Q. Hearn M. G. Pham D. G. (1996). cDNA Cloning and Chromosome Mapping of the Human Fe65 Gene: Interaction of the Conserved Cytoplasmic Domains of the Human Beta-Amyloid Precursor Protein and its Homologues with the Mouse Fe65 Protein. Hum. Mol. Genet. 5, 1589–1598. 10.1093/hmg/5.10.1589 8894693

[B39] Brum L. F. Elisabetsky E. Souza D. (2001). Effects of Linalool on [(3)H]MK801 and [(3)H] Muscimol Binding in Mouse Cortical Membranes. Phytother. Res. 15, 422–425. 10.1002/ptr.973 11507735

[B40] Burton M. D. Rytych J. L. Amin R. Johnson R. W. (2016). Dietary Luteolin Reduces Proinflammatory Microglia in the Brain of Senescent Mice. Rejuvenation Res. 19, 286–292. 10.1089/rej.2015.1708 26918466 PMC4971424

[B41] Busche M. A. Kekuš M. Adelsberger H. Noda T. Förstl H. Nelken I. (2015). Rescue of Long-Range Circuit Dysfunction in Alzheimer's Disease Models. Nat. Neurosci. 18, 1623–1630. 10.1038/nn.4137 26457554

[B42] Butterfield D. A. (2002). Amyloid Beta-Peptide (1-42)-induced Oxidative Stress and Neurotoxicity: Implications for Neurodegeneration in Alzheimer's Disease Brain. A Review. Free Radic. Res. 36, 1307–1313. 10.1080/1071576021000049890 12607822

[B43] Butterfield D. A. Boyd-Kimball D. (2018). Oxidative Stress, Amyloid-β Peptide, and Altered Key Molecular Pathways in the Pathogenesis and Progression of Alzheimer's Disease. J. Alzheimers Dis. 62, 1345–1367. 10.3233/JAD-170543 29562527 PMC5870019

[B44] Cai Q. Ma T. Li C. Tian Y. Li H. (2016). Catalpol Protects Pre-myelinating Oligodendrocytes against Ischemia-Induced Oxidative Injury through ERK1/2 Signaling Pathway. Int. J. Biol. Sci. 12, 1415–1426. 10.7150/ijbs.16823 27994507 PMC5166484

[B45] Cai X. Fang Z. Dou J. Yu A. Zhai G. (2013). Bioavailability of Quercetin: Problems and Promises. Curr. Med. Chem. 20, 2572–2582. 10.2174/09298673113209990120 23514412

[B46] Calvo-Rodriguez M. Hernando-Perez E. Nuñez L. Villalobos C. (2019). Amyloid β Oligomers Increase ER-Mitochondria Ca2+ Cross Talk in Young Hippocampal Neurons and Exacerbate Aging-Induced Intracellular Ca2+ Remodeling. Front. Cel. Neurosci. 13, 22. 10.3389/fncel.2019.00022 PMC6376150 30800057

[B47] Candelario-Jalil E. de Oliveira A. C. Gräf S. Bhatia H. S. Hüll M. Muñoz E. (2007). Resveratrol Potently Reduces Prostaglandin E2 Production and Free Radical Formation in Lipopolysaccharide-Activated Primary Rat Microglia. J. Neuroinflammation 4, 25. 10.1186/1742-2094-4-25 17927823 PMC2100038

[B48] Capiralla H. Vingtdeux V. Zhao H. Sankowski R. Al-Abed Y. Davies P. (2012). Resveratrol Mitigates Lipopolysaccharide- and Aβ-Mediated Microglial Inflammation by Inhibiting the TLR4/NF-Κb/STAT Signaling cascade. J. Neurochem. 120, 461–472. 10.1111/j.1471-4159.2011.07594.x 22118570 PMC3253186

[B49] Caputo L. Piccialli I. Ciccone R. de Caprariis P. Massa A. De Feo V. (2021). Lavender and Coriander Essential Oils and Their Main Component Linalool Exert a Protective Effect against Amyloid-β Neurotoxicity. Phytother. Res. 35, 486–493. 10.1002/ptr.6827 32785956

[B50] Casamenti F. Stefani M. (2017). Olive Polyphenols: New Promising Agents to Combat Aging-Associated Neurodegeneration. Expert Rev. Neurother. 17, 345–358. 10.1080/14737175.2017.1245617 27762153

[B51] Cerf E. Gustot A. Goormaghtigh E. Ruysschaert J. M. Raussens V. (2011). High Ability of Apolipoprotein E4 to Stabilize Amyloid-β Peptide Oligomers, the Pathological Entities Responsible for Alzheimer's Disease. FASEB J. 25, 1585–1595. 10.1096/fj.10-175976 21266538

[B52] Cheignon C. Tomas M. Bonnefont-Rousselot D. Faller P. Hureau C. Collin F. (2018). Oxidative Stress and the Amyloid Beta Peptide in Alzheimer's Disease. Redox Biol. 14, 450–464. 10.1016/j.redox.2017.10.014 29080524 PMC5680523

[B53] Chen C. H. Zhou W. Liu S. Deng Y. Cai F. Tone M. (2012). Increased NF-Κb Signalling Up-Regulates BACE1 Expression and its Therapeutic Potential in Alzheimer's Disease. Int. J. Neuropsychopharmacol. 15, 77–90. 10.1017/S1461145711000149 21329555

[B54] Chen G. F. Xu T. H. Yan Y. Zhou Y. R. Jiang Y. Melcher K. (2017). Amyloid Beta: Structure, Biology and Structure-Based Therapeutic Development. Acta Pharmacol. Sin. 38, 1205–1235. 10.1038/aps.2017.28 28713158 PMC5589967

[B55] Chen H. H. Liu P. Auger P. Lee S. H. Adolfsson O. Rey-Bellet L. (2018). Calpain-mediated Tau Fragmentation Is Altered in Alzheimer's Disease Progression. Sci. Rep. 8, 16725. 10.1038/s41598-018-35130-y 30425303 PMC6233188

[B56] Chen J. Yang Y. Lv Z. Shu A. Du Q. Wang W. (2020). Study on the Inhibitive Effect of Catalpol on Diabetic Nephropathy. Life Sci. 257, 118120. 10.1016/j.lfs.2020.118120 32693244

[B57] Chen Y. Shi G. W. Liang Z. M. Sheng S. Y. Shi Y. S. Peng L. (2019). Resveratrol Improves Cognition and Decreases Amyloid Plaque Formation in Tg6799 Mice. Mol. Med. Rep. 19, 3783–3790. 10.3892/mmr.2019.10010 30864708

[B58] Cheng-Chung Wei J. Huang H. C. Chen W. J. Huang C. N. Peng C. H. Lin C. L. (2016). Epigallocatechin Gallate Attenuates Amyloid β-induced Inflammation and Neurotoxicity in EOC 13.31 Microglia. Eur. J. Pharmacol. 770, 16–24. 10.1016/j.ejphar.2015.11.048 26643169

[B59] Choi C. W. Choi Y. H. Cha M. R. Kim Y. S. Yon G. H. Hong K. S. (2011). *In Vitro* BACE-1 Inhibitory Activity of Resveratrol Oligomers from the Seed Extract of Paeonia Lactiflora. Planta Med. 77, 374–376. 10.1055/s-0030-1250370 20890809

[B60] Chuengsamarn S. Rattanamongkolgul S. Luechapudiporn R. Phisalaphong C. Jirawatnotai S. (2012). Curcumin Extract for Prevention of Type 2 Diabetes. Diabetes Care 35, 2121–2127. 10.2337/dc12-0116 22773702 PMC3476912

[B61] Cichon N. Dziedzic A. Gorniak L. Miller E. Bijak M. Starosta M. (2021). Unusual Bioactive Compounds with Antioxidant Properties in Adjuvant Therapy Supporting Cognition Impairment in Age-Related Neurodegenerative Disorders. Int. J. Mol. Sci. 22, 10707. 10.3390/ijms221910707 34639048 PMC8509433

[B62] Comalada M. Ballester I. Bailón E. Sierra S. Xaus J. Gálvez J. (2006). Inhibition of Pro-inflammatory Markers in Primary Bone Marrow-Derived Mouse Macrophages by Naturally Occurring Flavonoids: Analysis of the Structure-Activity Relationship. Biochem. Pharmacol. 72, 1010–1021. 10.1016/j.bcp.2006.07.016 16934226

[B63] Cordaro M. Cuzzocrea S. Crupi R. (2020). An Update of Palmitoylethanolamide and Luteolin Effects in Preclinical and Clinical Studies of Neuroinflammatory Events. Antioxidants (Basel) 9, 216. 10.3390/antiox9030216 PMC7139331 32150935

[B64] Cunnane S. Nugent S. Roy M. Courchesne-Loyer A. Croteau E. Tremblay S. (2011). Brain Fuel Metabolism, Aging, and Alzheimer's Disease. Nutrition 27, 3–20. 10.1016/j.nut.2010.07.021 21035308 PMC3478067

[B65] d'Alessio P. A. Ostan R. Bisson J. F. Schulzke J. D. Ursini M. V. Béné M. C. (2013). Oral Administration of D-Limonene Controls Inflammation in Rat Colitis and Displays Anti-inflammatory Properties as Diet Supplementation in Humans. Life Sci. 92, 1151–1156. 10.1016/j.lfs.2013.04.013 23665426

[B66] Daily J. W. Kang S. Park S. (2021). Protection against Alzheimer's Disease by Luteolin: Role of Brain Glucose Regulation, Anti-inflammatory Activity, and the Gut Microbiota-Liver-Brain axis. Biofactors 47, 218–231. 10.1002/biof.1703 33347668

[B67] David D. C. Hauptmann S. Scherping I. Schuessel K. Keil U. Rizzu P. (2005). Proteomic and Functional Analyses Reveal a Mitochondrial Dysfunction in P301L Tau Transgenic Mice. J. Biol. Chem. 280, 23802–23814. 10.1074/jbc.M500356200 15831501

[B68] de la Monte S. M. (2017). Insulin Resistance and Neurodegeneration: Progress towards the Development of New Therapeutics for Alzheimer's Disease. Drugs 77, 47–65. 10.1007/s40265-016-0674-0 27988872 PMC5575843

[B69] de Oliveira J. Kucharska E. Garcez M. L. Rodrigues M. S. Quevedo J. Moreno-Gonzalez I. (2021). Inflammatory Cascade in Alzheimer's Disease Pathogenesis: A Review of Experimental Findings. Cells 10, 2581. 10.3390/cells10102581 34685563 PMC8533897

[B70] De Souza C. T. Araujo E. P. Bordin S. Ashimine R. Zollner R. L. Boschero A. C. (2005). Consumption of a Fat-Rich Diet Activates a Proinflammatory Response and Induces Insulin Resistance in the Hypothalamus. Endocrinology 146, 4192–4199. 10.1210/en.2004-1520 16002529

[B71] De Strooper B. Karran E. (2016). The Cellular Phase of Alzheimer's Disease. Cell 164, 603–615. 10.1016/j.cell.2015.12.056 26871627

[B72] DeFronzo R. A. Ferrannini E. Groop L. Henry R. R. Herman W. H. Holst J. J. (2015). Type 2 Diabetes Mellitus. Nat. Rev. Dis. Primers 1, 15019. 10.1038/nrdp.2015.19 27189025

[B73] Denzer I. Münch G. Friedland K. (2016). Modulation of Mitochondrial Dysfunction in Neurodegenerative Diseases via Activation of Nuclear Factor Erythroid-2-Related Factor 2 by Food-Derived Compounds. Pharmacol. Res. 103, 80–94. 10.1016/j.phrs.2015.11.019 26626189

[B74] Deqiu Z. Kang L. Jiali Y. Baolin L. Gaolin L. (2011). Luteolin Inhibits Inflammatory Response and Improves Insulin Sensitivity in the Endothelium. Biochimie 93, 506–512. 10.1016/j.biochi.2010.11.002 21081149

[B75] Devi S. Kumar V. Singh S. K. Dubey A. K. Kim J. J. (2021). Flavonoids: Potential Candidates for the Treatment of Neurodegenerative Disorders. Biomedicines 9, 99. 10.3390/biomedicines9020099 33498503 PMC7909525

[B76] Dhapola R. Hota S. S. Sarma P. Bhattacharyya A. Medhi B. Reddy D. H. (2021). Recent Advances in Molecular Pathways and Therapeutic Implications Targeting Neuroinflammation for Alzheimer's Disease. Inflammopharmacology 29, 1669–1681. 10.1007/s10787-021-00889-6 34813026 PMC8608577

[B77] Ding L. Jin D. Chen X. (2010). Luteolin Enhances Insulin Sensitivity via Activation of PPARγ Transcriptional Activity in Adipocytes. J. Nutr. Biochem. 21, 941–947. 10.1016/j.jnutbio.2009.07.009 19954946

[B78] Dirscherl K. Karlstetter M. Ebert S. Kraus D. Hlawatsch J. Walczak Y. (2010). Luteolin Triggers Global Changes in the Microglial Transcriptome Leading to a Unique Anti-inflammatory and Neuroprotective Phenotype. J. Neuroinflammation 7, 3. 10.1186/1742-2094-7-3 20074346 PMC2819254

[B79] Dominguez L. J. Veronese N. Vernuccio L. Catanese G. Inzerillo F. Salemi G. (2021). Nutrition, Physical Activity, and Other Lifestyle Factors in the Prevention of Cognitive Decline and Dementia. Nutrients 13, 4080. 10.3390/nu13114080 34836334 PMC8624903

[B80] Doytchinova I. Atanasova M. Salamanova E. Ivanov S. Dimitrov I. (2020). Curcumin Inhibits the Primary Nucleation of Amyloid-Beta Peptide: A Molecular Dynamics Study. Biomolecules 10, 1323. 10.3390/biom10091323 PMC7563689 32942739

[B81] Dubey M. Chaudhury P. Kabiru H. Shea T. B. (2008). Tau Inhibits Anterograde Axonal Transport and Perturbs Stability in Growing Axonal Neurites in Part by Displacing Kinesin Cargo: Neurofilaments Attenuate Tau-Mediated Neurite Instability. Cell. Motil. Cytoskeleton. 65, 89–99. 10.1002/cm.20243 18000878

[B82] Dujardin S. Hyman B. T. (2019). Tau Prion-like Propagation: State of the Art and Current Challenges. Adv. Exp. Med. Biol. 1184, 305–325. 10.1007/978-981-32-9358-8_23 32096046

[B83] Dursun E. Gezen-Ak D. Hanağası H. Bilgiç B. Lohmann E. Ertan S. (2015). The Interleukin 1 Alpha, Interleukin 1 Beta, Interleukin 6 and Alpha-2-Macroglobulin Serum Levels in Patients with Early or Late Onset Alzheimer's Disease, Mild Cognitive Impairment or Parkinson's Disease. J. Neuroimmunol. 283, 50–57. 10.1016/j.jneuroim.2015.04.014 26004156

[B84] Eddin L. B. Jha N. K. Meeran M. F. N. Kesari K. K. Beiram R. Ojha S. (2021). Neuroprotective Potential of Limonene and Limonene Containing Natural Products. Molecules 26, 4535. 10.3390/molecules26154535 34361686 PMC8348102

[B85] Efthymiou A. G. Goate A. M. (2017). Late Onset Alzheimer's Disease Genetics Implicates Microglial Pathways in Disease Risk. Mol. Neurodegener. 12, 43. 10.1186/s13024-017-0184-x 28549481 PMC5446752

[B86] Ehrnhoefer D. E. Bieschke J. Boeddrich A. Herbst M. Masino L. Lurz R. (2008). EGCG Redirects Amyloidogenic Polypeptides into Unstructured, Off-Pathway Oligomers. Nat. Struct. Mol. Biol. 15, 558–566. 10.1038/nsmb.1437 18511942

[B87] Engin A. B. (2017). What Is Lipotoxicity? Adv. Exp. Med. Biol. 960, 197–220. 10.1007/978-3-319-48382-5_8 28585200

[B88] Esteras N. Abramov A. Y. (2020). Mitochondrial Calcium Deregulation in the Mechanism of Beta-Amyloid and Tau Pathology. Cells 9, 2135. 10.3390/cells9092135 PMC7564294 32967303

[B89] Ettcheto M. Cano A. Manzine P. R. Busquets O. Verdaguer E. Castro-Torres R. D. (2020). Epigallocatechin-3-Gallate (EGCG) Improves Cognitive Deficits Aggravated by an Obesogenic Diet through Modulation of Unfolded Protein Response in APPswe/PS1dE9 Mice. Mol. Neurobiol. 57, 1814–1827. 10.1007/s12035-019-01849-6 31838720

[B90] Fan Q. Liu Y. Wang X. Zhang Z. Fu Y. Liu L. (2020). Ginnalin A Inhibits Aggregation, Reverses Fibrillogenesis, and Alleviates Cytotoxicity of Amyloid β(1-42). ACS Chem. Neurosci. 11, 638–647. 10.1021/acschemneuro.9b00673 31967782

[B91] Feng Y. Wang X. (2012). Antioxidant Therapies for Alzheimer's Disease. Oxid. Med. Cel. Longev. 2012, 472932. 10.1155/2012/472932 PMC3410354 22888398

[B92] Fernandez M. A. Klutkowski J. A. Freret T. Wolfe M. S. (2014). Alzheimer Presenilin-1 Mutations Dramatically Reduce Trimming of Long Amyloid β-peptides (Aβ) by γ-secretase to Increase 42-To-40-Residue Aβ. J. Biol. Chem. 289, 31043–31052. 10.1074/jbc.M114.581165 25239621 PMC4223309

[B93] Folch J. Ettcheto M. Petrov D. Abad S. Pedrós I. Marin M. (2018). Review of the Advances in Treatment for Alzheimer Disease: Strategies for Combating β-amyloid Protein. Neurologia 33, 47–58. 10.1016/j.nrl.2015.03.012 25976937

[B94] Forman H. J. Zhang H. (2021). Targeting Oxidative Stress in Disease: Promise and Limitations of Antioxidant Therapy. Nat. Rev. Drug Discov. 20, 689–709. 10.1038/s41573-021-00233-1 34194012 PMC8243062

[B95] Fu Z. Aucoin D. Ahmed M. Ziliox M. Van Nostrand W. E. Smith S. O. (2014). Capping of Aβ42 Oligomers by Small Molecule Inhibitors. Biochemistry 53, 7893–7903. 10.1021/bi500910b 25422864 PMC4278677

[B96] Fukutomi R. Ohishi T. Koyama Y. Pervin M. Nakamura Y. Isemura M. (2021). Beneficial Effects of Epigallocatechin-3-O-Gallate, Chlorogenic Acid, Resveratrol, and Curcumin on Neurodegenerative Diseases. Molecules 26, 415. 10.3390/molecules26020415 PMC7829779 33466849

[B97] Fusco D. Colloca G. Lo Monaco M. R. Cesari M. (2007). Effects of Antioxidant Supplementation on the Aging Process. Clin. Interv. Aging 2, 377–387. 18044188 PMC2685276

[B98] Gamblin T. C. Chen F. Zambrano A. Abraha A. Lagalwar S. Guillozet A. L. (2003). Caspase Cleavage of Tau: Linking Amyloid and Neurofibrillary Tangles in Alzheimer's Disease. Proc. Natl. Acad. Sci. U. S. A. 100, 10032–10037. 10.1073/pnas.1630428100 12888622 PMC187753

[B99] Gao C. Liu Y. Jiang Y. Ding J. Li L. (2014). Geniposide Ameliorates Learning Memory Deficits, Reduces Tau Phosphorylation and Decreases Apoptosis via GSK3β Pathway in Streptozotocin-Induced Alzheimer Rat Model. Brain Pathol. 24, 261–269. 10.1111/bpa.12116 24329968 PMC8029432

[B100] Gao Y. Tan L. Yu J. T. Tan L. (2018). Tau in Alzheimer's Disease: Mechanisms and Therapeutic Strategies. Curr. Alzheimer Res. 15, 283–300. 10.2174/1567205014666170417111859 28413986

[B101] Garcia-Alloza M. Borrelli L. A. Rozkalne A. Hyman B. T. Bacskai B. J. (2007). Curcumin Labels Amyloid Pathology *In Vivo*, Disrupts Existing Plaques, and Partially Restores Distorted Neurites in an Alzheimer Mouse Model. J. Neurochem. 102, 1095–1104. 10.1111/j.1471-4159.2007.04613.x 17472706

[B102] George-Hyslop P. S. Rossor M. (2001). Alzheimer's Disease. Unravelling the Disease Process. Lancet 358 (Suppl. l), S1. 10.1016/s0140-6736(01)07014-3 11784550

[B103] Ghobeh M. Ahmadian S. Meratan A. A. Ebrahim-Habibi A. Ghasemi A. Shafizadeh M. (2014). Interaction of Aβ(25-35) Fibrillation Products with Mitochondria: Effect of Small-Molecule Natural Products. Biopolymers 102, 473–486. 10.1002/bip.22572 25297917

[B104] Giraldo E. Lloret A. Fuchsberger T. Viña J. (2014). Aβ and Tau Toxicities in Alzheimer's Are Linked via Oxidative Stress-Induced P38 Activation: Protective Role of Vitamin E. Redox Biol. 2, 873–877. 10.1016/j.redox.2014.03.002 25061569 PMC4099506

[B105] Gong D. Liu B. Tan X. (2015). Genistein Prevents Cadmium-Induced Neurotoxic Effects through its Antioxidant Mechanisms. Drug Res. 65, 65–69. 10.1055/s-0034-1372595 24918346

[B106] Goodson H. V. Jonasson E. M. (2018). Microtubules and Microtubule-Associated Proteins. Cold Spring Harb. Perspect. Biol. 10, a022608. 10.1101/cshperspect.a022608 PMC5983186 29858272

[B107] Grosso G. (2018). Effects of Polyphenol-Rich Foods on Human Health. Nutrients 10. 10.3390/nu10081089 PMC6115785 30110959

[B108] Gu Z. Liu W. Yan Z. (2009). {beta}-Amyloid Impairs AMPA Receptor Trafficking and Function by Reducing Ca2+/calmodulin-dependent Protein Kinase II Synaptic Distribution. J. Biol. Chem. 284, 10639–10649. 10.1074/jbc.M806508200 19240035 PMC2667751

[B109] Guan R. Van Le Q. Yang H. Zhang D. Gu H. Yang Y. (2021). A Review of Dietary Phytochemicals and Their Relation to Oxidative Stress and Human Diseases. Chemosphere 271, 129499. 10.1016/j.chemosphere.2020.129499 33445014

[B110] Guénette S. Y. (2003). Astrocytes: a Cellular Player in Abeta Clearance and Degradation. Trends Mol. Med. 9, 279–280. 10.1016/s1471-4914(03)00112-6 12900212

[B111] Hall J. E. do Carmo J. M. da Silva A. A. Wang Z. Hall M. E. (2015). Obesity-induced Hypertension: Interaction of Neurohumoral and Renal Mechanisms. Circ. Res. 116, 991–1006. 10.1161/CIRCRESAHA.116.305697 25767285 PMC4363087

[B112] Hamaguchi T. Ono K. Yamada M. (2010). REVIEW: Curcumin and Alzheimer's Disease. CNS Neurosci. Ther. 16, 285–297. 10.1111/j.1755-5949.2010.00147.x 20406252 PMC6493893

[B113] Hansen D. V. Hanson J. E. Sheng M. (2018). Microglia in Alzheimer's Disease. J. Cel. Biol. 217, 459–472. 10.1083/jcb.201709069 PMC5800817 29196460

[B114] Harder D. R. Zhang C. Gebremedhin D. (2002). Astrocytes Function in Matching Blood Flow to Metabolic Activity. News Physiol. Sci. 17, 27–31. 10.1152/physiologyonline.2002.17.1.27 11821533

[B115] Hardy J. (1997). Amyloid, the Presenilins and Alzheimer's Disease. Trends Neurosci. 20, 154–159. 10.1016/s0166-2236(96)01030-2 9106355

[B116] Hashimoto T. Serrano-Pozo A. Hori Y. Adams K. W. Takeda S. Banerji A. O. (2012). Apolipoprotein E, Especially Apolipoprotein E4, Increases the Oligomerization of Amyloid β Peptide. J. Neurosci. 32, 15181–15192. 10.1523/JNEUROSCI.1542-12.2012 23100439 PMC3493562

[B117] He L. Zhao R. Wang Y. Liu H. Wang X. (2021). Research Progress on Catalpol as Treatment for Atherosclerosis. Front. Pharmacol. 12, 716125. 10.3389/fphar.2021.716125 34326774 PMC8313760

[B118] Heinrich M. (2010). Galanthamine from Galanthus and Other Amaryllidaceae-Cchemistry and Biology Based on Traditional Use. Alkaloids Chem. Biol. 8, 157–165. 10.1016/s1099-4831(10)06804-5 20334038

[B119] Henríquez G. Gomez A. Guerrero E. Narayan M. (2020). Potential Role of Natural Polyphenols against Protein Aggregation Toxicity: *In Vitro*, *In Vivo*, and Clinical Studies. ACS Chem. Neurosci. 11, 2915–2934. 10.1021/acschemneuro.0c00381 32822152

[B120] Hesse R. Wahler A. Gummert P. Kirschmer S. Otto M. Tumani H. (2016). Decreased IL-8 Levels in CSF and Serum of AD Patients and Negative Correlation of MMSE and IL-1β. BMC Neurol. 16, 185. 10.1186/s12883-016-0707-z 27671345 PMC5037590

[B121] Hirohata M. Ono K. Takasaki J. Takahashi R. Ikeda T. Morinaga A. (2012). Anti-amyloidoGenic Effects of Soybean Isoflavones *In Vitro*: Fluorescence Spectroscopy Demonstrating Direct Binding to Aβ Monomers, Oligomers and Fibrils. Biochim. Biophys. Acta 1822, 1316–1324. 10.1016/j.bbadis.2012.05.006 22587837

[B122] Ho L. Qin W. Pompl P. N. Xiang Z. Wang J. Zhao Z. (2004). Diet-induced Insulin Resistance Promotes Amyloidosis in a Transgenic Mouse Model of Alzheimer's Disease. FASEB J. 18, 902–904. 10.1096/fj.03-0978fje 15033922

[B123] Hole K. L. Williams R. J. (2021). Flavonoids as an Intervention for Alzheimer's Disease: Progress and Hurdles towards Defining a Mechanism of Action. Brain Plast. 6, 167–192. 10.3233/BPL-200098 33782649 PMC7990465

[B124] Holtzman D. M. Morris J. C. Goate A. M. (2011). Alzheimer's Disease: the challenge of the Second century. Sci. Transl. Med. 3, 77sr1. 10.1126/scitranslmed.3002369 21471435 PMC3130546

[B125] Hong M. Lee V. M. (1997). Insulin and Insulin-like Growth Factor-1 Regulate Tau Phosphorylation in Cultured Human Neurons. J. Biol. Chem. 272, 19547–19553. 10.1074/jbc.272.31.19547 9235959

[B126] Hoover B. R. Reed M. N. Su J. Penrod R. D. Kotilinek L. A. Grant M. K. (2010). Tau Mislocalization to Dendritic Spines Mediates Synaptic Dysfunction Independently of Neurodegeneration. Neuron 68, 1067–1081. 10.1016/j.neuron.2010.11.030 21172610 PMC3026458

[B127] Huang J. Z. Wu J. Xiang S. Sheng S. Jiang Y. Yang Z. (2016). Catalpol Preserves Neural Function and Attenuates the Pathology of Alzheimer's Disease in Mice. Mol. Med. Rep. 13, 491–496. 10.3892/mmr.2015.4496 26531891

[B128] Huang W. J. Niu H. S. Lin M. H. Cheng J. T. Hsu F. L. (2010). Antihyperglycemic Effect of Catalpol in Streptozotocin-Induced Diabetic Rats. J. Nat. Prod. 73, 1170–1172. 10.1021/np9008317 20518543

[B129] Hughes R. H. Silva V. A. Ahmed I. Shreiber D. I. Morrison B. 3rd. (2014). Neuroprotection by Genipin against Reactive Oxygen and Reactive Nitrogen Species-Mediated Injury in Organotypic Hippocampal Slice Cultures. Brain Res. 1543, 308–314. 10.1016/j.brainres.2013.11.020 24275198

[B130] Hung C. H. Cheng S. S. Cheung Y. T. Wuwongse S. Zhang N. Q. Ho Y. S. (2018). A Reciprocal Relationship between Reactive Oxygen Species and Mitochondrial Dynamics in Neurodegeneration. Redox Biol. 14, 7–19. 10.1016/j.redox.2017.08.010 28837882 PMC5567977

[B131] Husna Ibrahim N. Yahaya M. F. Mohamed W. Teoh S. L. Hui C. K. Kumar J. (2020). Pharmacotherapy of Alzheimer's Disease: Seeking Clarity in a Time of Uncertainty. Front. Pharmacol. 11, 261. 10.3389/fphar.2020.00261 32265696 PMC7105678

[B132] Inoue K. Nakagawa A. Hino T. Oka H. (2009). Screening Assay for Metal-Catalyzed Oxidation Inhibitors Using Liquid Chromatography-Mass Spectrometry with an N-Terminal Beta-Amyloid Peptide. Anal. Chem. 81, 1819–1825. 10.1021/ac802162n 19173589

[B133] Ishige K. Schubert D. Sagara Y. (2001). Flavonoids Protect Neuronal Cells from Oxidative Stress by Three Distinct Mechanisms. Free Radic. Biol. Med. 30, 433–446. 10.1016/s0891-5849(00)00498-6 11182299

[B134] Janota C. Lemere C. A. Brito M. A. (2016). Dissecting the Contribution of Vascular Alterations and Aging to Alzheimer's Disease. Mol. Neurobiol. 53, 3793–3811. 10.1007/s12035-015-9319-7 26143259

[B135] Jeremic D. Jiménez-Díaz L. Navarro-López J. D. (2021). Past, Present and Future of Therapeutic Strategies against Amyloid-β Peptides in Alzheimer's Disease: a Systematic Review. Ageing Res. Rev. 72, 101496. 10.1016/j.arr.2021.101496 34687956

[B136] Jhamandas J. H. Cho C. Jassar B. Harris K. MacTavish D. Easaw J. (2001). Cellular Mechanisms for Amyloid Beta-Protein Activation of Rat Cholinergic Basal Forebrain Neurons. J. Neurophysiol. 86, 1312–1320. 10.1152/jn.2001.86.3.1312 11535679

[B137] Jiang B. Du J. Liu J. H. Bao Y. M. An L. J. (2008). Catalpol Attenuates the Neurotoxicity Induced by Beta-Amyloid(1-42) in Cortical Neuron-Glia Cultures. Brain Res. 1188, 139–147. 10.1016/j.brainres.2007.07.105 18022141

[B138] Jin X. Liu M. Y. Zhang D. F. Zhong X. Du K. Qian P. (2019). Natural Products as a Potential Modulator of Microglial Polarization in Neurodegenerative Diseases. Pharmacol. Res. 145, 104253. 10.1016/j.phrs.2019.104253 31059788

[B139] Jing L. Zhang Y. Fan S. Gu M. Guan Y. Lu X. (2013). Preventive and Ameliorating Effects of Citrus D-Limonene on Dyslipidemia and Hyperglycemia in Mice with High-Fat Diet-Induced Obesity. Eur. J. Pharmacol. 715, 46–55. 10.1016/j.ejphar.2013.06.022 23838456

[B140] Jurcau A. (2021). The Role of Natural Antioxidants in the Prevention of Dementia-Where Do We Stand and Future Perspectives. Nutrients 13, 282. 10.3390/nu13020282 33498262 PMC7909256

[B141] Kametani F. Hasegawa M. (2018). Reconsideration of Amyloid Hypothesis and Tau Hypothesis in Alzheimer's Disease. Front. Neurosci. 12, 25. 10.3389/fnins.2018.00025 29440986 PMC5797629

[B142] Kang C. H. Choi Y. H. Moon S. K. Kim W. J. Kim G. Y. (2013). Quercetin Inhibits Lipopolysaccharide-Induced Nitric Oxide Production in BV2 Microglial Cells by Suppressing the NF-Κb Pathway and Activating the Nrf2-dependent HO-1 Pathway. Int. Immunopharmacol. 17, 808–813. 10.1016/j.intimp.2013.09.009 24076371

[B143] Kapogiannis D. Mattson M. P. (2011). Disrupted Energy Metabolism and Neuronal Circuit Dysfunction in Cognitive Impairment and Alzheimer's Disease. Lancet Neurol. 10, 187–198. 10.1016/S1474-4422(10)70277-5 21147038 PMC3026092

[B144] Khaki A. Fathiazad F. Nouri M. Khaki A. Maleki N. A. Khamnei H. J. (2010). Beneficial Effects of Quercetin on Sperm Parameters in Streptozotocin-Induced Diabetic Male Rats. Phytother. Res. 24, 1285–1291. 10.1002/ptr.3100 20127875

[B145] Kheiri G. Dolatshahi M. Rahmani F. Rezaei N. (2018). Role of p38/MAPKs in Alzheimer's Disease: Implications for Amyloid Beta Toxicity Targeted Therapy. Rev. Neurosci. 30, 9–30. 10.1515/revneuro-2018-0008 29804103

[B146] Koffie R. M. Hashimoto T. Tai H. C. Kay K. R. Serrano-Pozo A. Joyner D. (2012). Apolipoprotein E4 Effects in Alzheimer's Disease Are Mediated by Synaptotoxic Oligomeric Amyloid-β. Brain 135, 2155–2168. 10.1093/brain/aws127 22637583 PMC3381721

[B147] Kou J. J. Shi J. Z. He Y. Y. Hao J. J. Zhang H. Y. Luo D. M. (2021). Luteolin Alleviates Cognitive Impairment in Alzheimer's Disease Mouse Model via Inhibiting Endoplasmic Reticulum Stress-dependent Neuroinflammation. Acta Pharmacol. Sin. 43, 1–10. 10.1038/s41401-021-00702-8 34267346 PMC8975883

[B148] Koukoulitsa C. Villalonga-Barber C. Csonka R. Alexi X. Leonis G. Dellis D. (2016). Biological and Computational Evaluation of Resveratrol Inhibitors against Alzheimer's Disease. J. Enzyme Inhib. Med. Chem. 31, 67–77. 10.3109/14756366.2014.1003928 26147348

[B149] Kowalik-Jankowska T. Ruta M. Wiśniewska K. Łankiewicz L. Dyba M. (2004). Products of Cu(II)-catalyzed Oxidation in the Presence of Hydrogen Peroxide of the 1-10, 1-16 Fragments of Human and Mouse Beta-Amyloid Peptide. J. Inorg. Biochem. 98, 940–950. 10.1016/j.jinorgbio.2004.03.001 15149800

[B150] Kumari A. Yadav S. K. Pakade Y. B. Singh B. Yadav S. C. (2010). Development of Biodegradable Nanoparticles for Delivery of Quercetin. Colloids Surf. B Biointerfaces 80, 184–192. 10.1016/j.colsurfb.2010.06.002 20598513

[B151] Kurochkin I. V. Guarnera E. Berezovsky I. N. (2018). Insulin-Degrading Enzyme in the Fight against Alzheimer's Disease. Trends Pharmacol. Sci. 39, 49–58. 10.1016/j.tips.2017.10.008 29132916

[B152] Kwok J. B. Loy C. T. Dobson-Stone C. Halliday G. M. (2020). The Complex Relationship between Genotype, Pathology and Phenotype in Familial Dementia. Neurobiol. Dis. 145, 105082. 10.1016/j.nbd.2020.105082 32927063

[B153] Laurent C. Buée L. Blum D. (2018). Tau and Neuroinflammation: What Impact for Alzheimer's Disease and Tauopathies? Biomed. J. 41, 21–33. 10.1016/j.bj.2018.01.003 29673549 PMC6138617

[B154] Lazar A. N. Mourtas S. Youssef I. Parizot C. Dauphin A. Delatour B. (2013). Curcumin-conjugated Nanoliposomes with High Affinity for Aβ Deposits: Possible Applications to Alzheimer Disease. Nanomedicine 9, 712–721. 10.1016/j.nano.2012.11.004 23220328

[B155] Lee C. W. Yen F. L. Huang H. W. Wu T. H. Ko H. H. Tzeng W. S. (2012). Resveratrol Nanoparticle System Improves Dissolution Properties and Enhances the Hepatoprotective Effect of Resveratrol through Antioxidant and Anti-inflammatory Pathways. J. Agric. Food Chem. 60, 4662–4671. 10.1021/jf2050137 22480310

[B156] Leloup C. Arluison M. Kassis N. Lepetit N. Cartier N. Ferré P. (1996). Discrete Brain Areas Express the Insulin-Responsive Glucose Transporter GLUT4. Brain Res. Mol. Brain Res. 38, 45–53. 10.1016/0169-328x(95)00306-d 8737666

[B157] Li C. C. Hsiang C. Y. Lo H. Y. Pai F. T. Wu S. L. Ho T. Y. (2012). Genipin Inhibits Lipopolysaccharide-Induced Acute Systemic Inflammation in Mice as Evidenced by Nuclear Factor-Κb Bioluminescent Imaging-Guided Transcriptomic Analysis. Food Chem. Toxicol. 50, 2978–2986. 10.1016/j.fct.2012.05.054 22687549

[B158] Li F. Zhan C. Dong X. Wei G. (2021). Molecular Mechanisms of Resveratrol and EGCG in the Inhibition of Aβ42 Aggregation and Disruption of Aβ42 Protofibril: Similarities and Differences. Phys. Chem. Chem. Phys. 23, 18843–18854. 10.1039/d1cp01913a 34612422

[B159] Li H. Cao L. Ren Y. Jiang Y. Xie W. Li D. (2018). GLP-1 Receptor Regulates Cell Growth through Regulating IDE Expression Level in Aβ1-42-Treated PC12 Cells. Biosci. Rep. 38, BSR20171284. 10.1042/BSR20171284 29263141 PMC6043719

[B160] Li J. M. Li Y. C. Kong L. D. Hu Q. H. (2010). Curcumin Inhibits Hepatic Protein-Tyrosine Phosphatase 1B and Prevents Hypertriglyceridemia and Hepatic Steatosis in Fructose-Fed Rats. Hepatology 51, 1555–1566. 10.1002/hep.23524 20222050

[B161] Li J. Wang F. Ding H. Jin C. Chen J. Zhao Y. (2014). Geniposide, the Component of the Chinese Herbal Formula Tongluojiunao, Protects Amyloid-β Peptide (1-42-mediated Death of Hippocampal Neurons via the Non-classical Estrogensignaling Pathway. Neural Regen. Res. 9, 474–480. 10.4103/1673-5374.130063 25206841 PMC4153512

[B162] Li M. Cai N. Gu L. Yao L. Bi D. Fang W. (2021). Genipin Attenuates Tau Phosphorylation and Aβ Levels in Cellular Models of Alzheimer's Disease. Mol. Neurobiol. 58, 4134–4144. 10.1007/s12035-021-02389-8 33948899

[B163] Li S. Selkoe D. J. (2020). A Mechanistic Hypothesis for the Impairment of Synaptic Plasticity by Soluble Aβ Oligomers from Alzheimer's Brain. J. Neurochem. 154, 583–597. 10.1111/jnc.15007 32180217 PMC7487043

[B164] Li Y. Li L. Hölscher C. (2016). Therapeutic Potential of Genipin in Central Neurodegenerative Diseases. CNS Drugs 30, 889–897. 10.1007/s40263-016-0369-9 27395402

[B165] Liang J. H. Du J. Xu L. D. Jiang T. Hao S. Bi J. (2009). Catalpol Protects Primary Cultured Cortical Neurons Induced by Abeta(1-42) through a Mitochondrial-dependent Caspase Pathway. Neurochem. Int. 55, 741–746. 10.1016/j.neuint.2009.07.004 19631247

[B166] Lin M. T. Simon D. K. Ahn C. H. Kim L. M. Beal M. F. (2002). High Aggregate burden of Somatic mtDNA point Mutations in Aging and Alzheimer’s Disease Brain. Hum. Mol. Genet. 11, 133–145. 10.1093/hmg/11.2.133 11809722

[B167] Liskowsky W. Schliebs R. (2006). Muscarinic Acetylcholine Receptor Inhibition in Transgenic Alzheimer-like Tg2576 Mice by Scopolamine Favours the Amyloidogenic Route of Processing of Amyloid Precursor Protein. Int. J. Dev. Neurosci. 24, 149–156. 10.1016/j.ijdevneu.2005.11.010 16423497

[B168] Liu C. Chen K. Lu Y. Fang Z. Yu G. (2018). Catalpol Provides a Protective Effect on Fibrillary Aβ1-42 -induced Barrier Disruption in an *In Vitro* Model of the Blood-Brain Barrier. Phytother. Res. 32, 1047–1055. 10.1002/ptr.6043 29479743

[B169] Liu G. H. Qu J. Shen X. (2008). NF-kappaB/p65 Antagonizes Nrf2-ARE Pathway by Depriving CBP from Nrf2 and Facilitating Recruitment of HDAC3 to MafK. Biochim. Biophys. Acta 1783, 713–727. 10.1016/j.bbamcr.2008.01.002 18241676

[B170] Liu J. H. Yin F. Guo L. X. Deng X. H. Hu Y. H. (2009). Neuroprotection of Geniposide against Hydrogen Peroxide Induced PC12 Cells Injury: Involvement of PI3 Kinase Signal Pathway. Acta Pharmacol. Sin. 30, 159–165. 10.1038/aps.2008.25 19151742 PMC4002468

[B171] Liu J. Liu Z. Zhang Y. Yin F. (2015). Leptin Signaling Plays a Critical Role in the Geniposide-Induced Decrease of Tau Phosphorylation. Acta Biochim. Biophys. Sin. (Shanghai) 47, 1018–1022. 10.1093/abbs/gmv106 26496899

[B172] Liu J. Yin F. Xiao H. Guo L. Gao X. (2012). Glucagon-like Peptide 1 Receptor Plays an Essential Role in Geniposide Attenuating Lipotoxicity-Induced β-cell Apoptosis. Toxicol. Vitro 26, 1093–1097. 10.1016/j.tiv.2012.07.004 22819839

[B173] Liu J. Yin F. Zheng X. Jing J. Hu Y. (2007). Geniposide, a Novel Agonist for GLP-1 Receptor, Prevents PC12 Cells from Oxidative Damage via MAP Kinase Pathway. Neurochem. Int. 51, 361–369. 10.1016/j.neuint.2007.04.021 17629357

[B174] Liu J. Y. Zheng C. Z. Hao X. P. Zhang D. J. Mao A. W. Yuan P. (2016). Catalpol Ameliorates Diabetic Atherosclerosis in Diabetic Rabbits. Am. J. Transl. Res. 8, 4278–4288. 27830011 PMC5095320

[B175] Liu J. Zhang Y. Deng X. Yin F. (2013). Geniposide Decreases the Level of Aβ1-42 in the hippocampus of Streptozotocin-Induced Diabetic Rats. Acta Biochim. Biophys. Sin. (Shanghai) 45, 787–791. 10.1093/abbs/gmt069 23803411

[B176] Liu J. Zheng X. Yin F. Hu Y. Guo L. Deng X. (2006). Neurotrophic Property of Geniposide for Inducing the Neuronal Differentiation of PC12 Cells. Int. J. Dev. Neurosci. 24, 419–424. 10.1016/j.ijdevneu.2006.08.009 17045447

[B177] Liu Y. Fu X. Lan N. Li S. Zhang J. Wang S. (2014). Luteolin Protects against High Fat Diet-Induced Cognitive Deficits in Obesity Mice. Behav. Brain Res. 267, 178–188. 10.1016/j.bbr.2014.02.040 24667364

[B178] Liu Z. Zhang Y. Liu J. Yin F. (2017). Geniposide Attenuates the Level of Aβ1-42 via Enhancing Leptin Signaling in Cellular and APP/PS1 Transgenic Mice. Arch. Pharm. Res. 40, 571–578. 10.1007/s12272-016-0875-9 28160136

[B179] Long J. M. Holtzman D. M. (2019). Alzheimer Disease: An Update on Pathobiology and Treatment Strategies. Cell 179, 312–339. 10.1016/j.cell.2019.09.001 31564456 PMC6778042

[B180] Lu C. Guo Y. Yan J. Luo Z. Luo H. B. Yan M. (2013). Design, Synthesis, and Evaluation of Multitarget-Directed Resveratrol Derivatives for the Treatment of Alzheimer's Disease. J. Med. Chem. 56, 5843–5859. 10.1021/jm400567s 23799643

[B181] Lu X. Ma L. Ruan L. Kong Y. Mou H. Zhang Z. (2010). Resveratrol Differentially Modulates Inflammatory Responses of Microglia and Astrocytes. J. Neuroinflammation 7, 46. 10.1186/1742-2094-7-46 20712904 PMC2936301

[B182] Lv C. Liu X. Liu H. Chen T. Zhang W. (2014). Geniposide Attenuates Mitochondrial Dysfunction and Memory Deficits in APP/PS1 Transgenic Mice. Curr. Alzheimer Res. 11, 580–587. 10.2174/1567205011666140618095925 25034042

[B183] Lv M. Yang S. Cai L. Qin L. Q. Li B. Y. Wan Z. (2018). Effects of Quercetin Intervention on Cognition Function in APP/PS1 Mice Was Affected by Vitamin D Status. Mol. Nutr. Food Res. 62, e1800621. 10.1002/mnfr.201800621 30328681

[B184] Lye S. Aust C. E. Griffiths L. R. Fernandez F. (2021). Exploring New Avenues for Modifying Course of Progression of Alzheimer's Disease: The Rise of Natural Medicine. J. Neurol. Sci. 422, 117332. 10.1016/j.jns.2021.117332 33607542

[B185] Ma L. Yang C. Zheng J. Chen Y. Xiao Y. Huang K. (2020). Non-polyphenolic Natural Inhibitors of Amyloid Aggregation. Eur. J. Med. Chem. 192, 112197. 10.1016/j.ejmech.2020.112197 32172082

[B186] Mandelkow E. von Bergen M. Biernat J. Mandelkow E. M. (2007). Structural Principles of Tau and the Paired Helical Filaments of Alzheimer's Disease. Brain Pathol. 17, 83–90. 10.1111/j.1750-3639.2007.00053.x 17493042 PMC8095506

[B187] Mao Y. R. Jiang L. Duan Y. L. An L. J. Jiang B. (2007). Efficacy of Catalpol as Protectant against Oxidative Stress and Mitochondrial Dysfunction on Rotenone-Induced Toxicity in Mice Brain. Environ. Toxicol. Pharmacol. 23, 314–318. 10.1016/j.etap.2006.11.012 21783774

[B188] Marchi S. Giorgi C. Suski J. M. Agnoletto C. Bononi A. Bonora M. (2012). Mitochondria-ros Crosstalk in the Control of Cell Death and Aging. J. Signal. Transduct. 329635. 10.1155/2012/329635 PMC3235816 22175013

[B189] Marín-Royo G. Rodríguez C. Le Pape A. Jurado-López R. Luaces M. Antequera A. (2019). The Role of Mitochondrial Oxidative Stress in the Metabolic Alterations in Diet-Induced Obesity in Rats. FASEB J. 33, 12060–12072. 10.1096/fj.201900347RR 31370681 PMC6902682

[B190] Martin L. Latypova X. Wilson C. M. Magnaudeix A. Perrin M. L. Yardin C. (2013). Tau Protein Kinases: Involvement in Alzheimer's Disease. Ageing Res. Rev. 12, 289–309. 10.1016/j.arr.2012.06.003 22742992

[B191] Marucci G. Buccioni M. Ben D. D. Lambertucci C. Volpini R. Amenta F. (2021). Efficacy of Acetylcholinesterase Inhibitors in Alzheimer's Disease. Neuropharmacology 190, 108352. 10.1016/j.neuropharm.2020.108352 33035532

[B192] Matsuzaki K. Noguch T. Wakabayashi M. Ikeda K. Okada T. Ohashi Y. (2007). Inhibitors of Amyloid Beta-Protein Aggregation Mediated by GM1-Containing Raft-like Membranes. Biochim. Biophys. Acta 1768, 122–130. 10.1016/j.bbamem.2006.09.014 17069749

[B193] McCarty M. F. DiNicolantonio J. J. Lerner A. (2021). A Fundamental Role for Oxidants and Intracellular Calcium Signals in Alzheimer's Pathogenesis-And How a Comprehensive Antioxidant Strategy May Aid Prevention of This Disorder. Int. J. Mol. Sci. 22, 2140. 10.3390/ijms22042140 33669995 PMC7926325

[B194] Méndez-del Villar M. González-Ortiz M. Martínez-Abundis E. Pérez-Rubio K. G. Lizárraga-Valdez R. (2014). Effect of Resveratrol Administration on Metabolic Syndrome, Insulin Sensitivity, and Insulin Secretion. Metab. Syndr. Relat. Disord. 12, 497–501. 10.1089/met.2014.0082 25137036

[B195] Milstein J. L. Ferris H. A. (2021). The Brain as an Insulin-Sensitive Metabolic Organ. Mol. Metab. 52, 101234. 10.1016/j.molmet.2021.101234 33845179 PMC8513144

[B196] Miners J. S. Barua N. Kehoe P. G. Gill S. Love S. (2011). Aβ-degrading Enzymes: Potential for Treatment of Alzheimer Disease. J. Neuropathol. Exp. Neurol. 70, 944–959. 10.1097/NEN.0b013e3182345e46 22002425

[B197] Miranda A. Montiel E. Ulrich H. Paz C. (2021). Selective Secretase Targeting for Alzheimer's Disease Therapy. J. Alzheimers Dis. 81, 1–17. 10.3233/JAD-201027 33749645

[B198] Misonou H. Morishima-Kawashima M. Ihara Y. (2000). Oxidative Stress Induces Intracellular Accumulation of Amyloid Beta-Protein (Abeta) in Human Neuroblastoma Cells. Biochemistry 39, 6951–6959. 10.1021/bi000169p 10841777

[B199] Mithu V. S. Sarkar B. Bhowmik D. Das A. K. Chandrakesan M. Maiti S. (2014). Curcumin Alters the Salt Bridge-Containing Turn Region in Amyloid β(1-42) Aggregates. J. Biol. Chem. 289, 11122–11131. 10.1074/jbc.M113.519447 24599958 PMC4036251

[B200] Molaei A. Hatami H. Dehghan G. Sadeghian R. Khajehnasiri N. (2020). Synergistic Effects of Quercetin and Regular Exercise on the Recovery of Spatial Memory and Reduction of Parameters of Oxidative Stress in Animal Model of Alzheimer's Disease. EXCLI J. 19, 596–612. 10.17179/excli2019-2082 32483406 PMC7257248

[B201] Morales I. Cerda-Troncoso C. Andrade V. Maccioni R. B. (2017). The Natural Product Curcumin as a Potential Coadjuvant in Alzheimer's Treatment. J. Alzheimers Dis. 60, 451–460. 10.3233/JAD-170354 28854504

[B202] Morris J. C. Storandt M. McKeel D. W. Jr Rubin E. H. Price J. L. Grant E. A. (1996). Cerebral Amyloid Deposition and Diffuse Plaques in "normal" Aging: Evidence for Presymptomatic and Very Mild Alzheimer's Disease. Neurology 46, 707–719. 10.1212/wnl.46.3.707 8618671

[B203] Mourtas S. Lazar A. N. Markoutsa E. Duyckaerts C. Antimisiaris S. G. (2014). Multifunctional Nanoliposomes with Curcumin-Lipid Derivative and Brain Targeting Functionality with Potential Applications for Alzheimer Disease. Eur. J. Med. Chem. 80, 175–183. 10.1016/j.ejmech.2014.04.050 24780594

[B204] Moussa C. Hebron M. Huang X. Ahn J. Rissman R. A. Aisen P. S. (2017). Resveratrol Regulates Neuro-Inflammation and Induces Adaptive Immunity in Alzheimer's Disease. J. Neuroinflammation 14, 1. 10.1186/s12974-016-0779-0 28086917 PMC5234138

[B205] Murali R. Karthikeyan A. Saravanan R. (2013). Protective Effects of D-Limonene on Lipid Peroxidation and Antioxidant Enzymes in Streptozotocin-Induced Diabetic Rats. Basic Clin. Pharmacol. Toxicol. 112, 175–181. 10.1111/bcpt.12010 22998493

[B206] Nair S. Doh S. T. Chan J. Y. Kong A. N. Cai L. (2008). Regulatory Potential for Concerted Modulation of Nrf2- and Nfkb1-Mediated Gene Expression in Inflammation and Carcinogenesis. Br. J. Cancer 99, 2070–2082. 10.1038/sj.bjc.6604703 19050705 PMC2607222

[B207] Najafabadi R. E. Kazemipour N. Esmaeili A. Beheshti S. Nazifi S. (2018). Quercetin Prevents Body Weight Loss Due to the Using of Superparamagnetic Iron Oxide Nanoparticles in Rat. Adv. Biomed. Res. 7, 8. 10.4103/abr.abr_141_17 29456979 PMC5812102

[B208] Najarzadeh Z. Mohammad-Beigi H. Nedergaard Pedersen J. Christiansen G. Sønderby T. V. Shojaosadati S. A. (2019). Plant Polyphenols Inhibit Functional Amyloid and Biofilm Formation in Pseudomonas Strains by Directing Monomers to Off-Pathway Oligomers. Biomolecules 9, 659. 10.3390/biom9110659 PMC6920965 31717821

[B209] Näslund J. Schierhorn A. Hellman U. Lannfelt L. Roses A. D. Tjernberg L. O. (1994). Relative Abundance of Alzheimer A Beta Amyloid Peptide Variants in Alzheimer Disease and normal Aging. Proc. Natl. Acad. Sci. U. S. A. 91, 8378–8382. 10.1073/pnas.91.18.8378 8078890 PMC44609

[B210] Neumann U. Ufer M. Jacobson L. H. Rouzade-Dominguez M. L. Huledal G. Kolly C. (2018). The BACE-1 Inhibitor CNP520 for Prevention Trials in Alzheimer's Disease. EMBO Mol. Med. 10, e9316. 10.15252/emmm.201809316 30224383 PMC6220303

[B211] Nissanka N. Moraes C. T. (2018). Mitochondrial DNA Damage and Reactive Oxygen Species in Neurodegenerative Disease. FEBS Lett. 592, 728–742. 10.1002/1873-3468.12956 29281123 PMC6942696

[B212] Olajide O. A. Sarker S. D. (2020). Alzheimer's Disease: Natural Products as Inhibitors of Neuroinflammation. Inflammopharmacology 28, 1439–1455. 10.1007/s10787-020-00751-1 32930914 PMC7572326

[B213] Ono K. Hasegawa K. Naiki H. Yamada M. (2004). Curcumin Has Potent Anti-amyloidogenic Effects for Alzheimer's Beta-Amyloid Fibrils *In Vitro* . J. Neurosci. Res. 75, 742–750. 10.1002/jnr.20025 14994335

[B214] Ortsäter H. Grankvist N. Wolfram S. Kuehn N. Sjöholm A. (2012). Diet Supplementation with green tea Extract Epigallocatechin Gallate Prevents Progression to Glucose Intolerance in Db/db Mice. Nutr. Metab. (Lond.) 9, 11. 10.1186/1743-7075-9-11 22333133 PMC3298777

[B215] Oset-Gasque M. J. Marco-Contelles J. (2018). Alzheimer's Disease, the "One-Molecule, One-Target" Paradigm, and the Multitarget Directed Ligand Approach. ACS Chem. Neurosci. 9, 401–403. 10.1021/acschemneuro.8b00069 29465220

[B216] Pallauf K. Duckstein N. Hasler M. Klotz L. O. Rimbach G. (2017). Flavonoids as Putative Inducers of the Transcription Factors Nrf2, FoxO, and PPARgamma. Oxid. Med. Cel. Longev. 4397340. 10.1155/2017/4397340 PMC5518529 28761622

[B217] Palop J. J. Chin J. Roberson E. D. Wang J. Thwin M. T. Bien-Ly N. (2007). Aberrant Excitatory Neuronal Activity and Compensatory Remodeling of Inhibitory Hippocampal Circuits in Mouse Models of Alzheimer's Disease. Neuron 55, 697–711. 10.1016/j.neuron.2007.07.025 17785178 PMC8055171

[B218] Pannaccione A. Boscia F. Scorziello A. Adornetto A. Castaldo P. Sirabella R. (2007). Up-regulation and Increased Activity of KV3.4 Channels and Their Accessory Subunit MinK-Related Peptide 2 Induced by Amyloid Peptide Are Involved in Apoptotic Neuronal Death. Mol. Pharmacol. 72, 665–673. 10.1124/mol.107.034868 17495071

[B219] Pannaccione A. Piccialli I. Secondo A. Ciccone R. Molinaro P. Boscia F. (2020). The Na+/Ca2+exchanger in Alzheimer's Disease. Cell Calcium 87, 102190. 10.1016/j.ceca.2020.102190 32199208

[B220] Park S. Kim D. S. Kang S. Kim H. J. (2018). The Combination of Luteolin and L-Theanine Improved Alzheimer Disease-like Symptoms by Potentiating Hippocampal Insulin Signaling and Decreasing Neuroinflammation and Norepinephrine Degradation in Amyloid-β-Infused Rats. Nutr. Res. 60, 116–131. 10.1016/j.nutres.2018.09.010 30527255

[B221] Park S. Y. Kim H. S. Cho E. K. Kwon B. Y. Phark S. Hwang K. W. (2008). Curcumin Protected PC12 Cells against Beta-Amyloid-Induced Toxicity through the Inhibition of Oxidative Damage and Tau Hyperphosphorylation. Food Chem. Toxicol. 46, 2881–2887. 10.1016/j.fct.2008.05.030 18573304

[B222] Paterniti I. Cordaro M. Campolo M. Siracusa R. Cornelius C. Navarra M. (2014). Neuroprotection by Association of Palmitoylethanolamide with Luteolin in Experimental Alzheimer's Disease Models: the Control of Neuroinflammation. CNS Neurol. Disord. Drug Targets 13, 1530–1541. 10.2174/1871527313666140806124322 25106636

[B223] Pedersen W. A. Kloczewiak M. A. Blusztajn J. K. (1996). Amyloid β-protein Reduces Acetylcholine Synthesis in a Cell Line Derived from Cholinergic Neurons of the Basal Forebrain. Proc. Natl. Acad. Sci. U. S. A. 93, 8068–8071. 10.1073/pnas.93.15.8068 8755604 PMC38876

[B224] Peña F. Gutiérrez-Lerma A. Quiroz-Baez R. Arias C. (2006). The Role of Beta-Amyloid Protein in Synaptic Function: Implications for Alzheimer's Disease Therapy. Curr. Neuropharmacol. 4, 149–163. 10.2174/157015906776359531 18615129 PMC2430670

[B225] Perea J. R. Ávila J. Bolós M. (2018). Dephosphorylated rather Than Hyperphosphorylated Tau Triggers a Pro-inflammatory Profile in Microglia through the P38 MAPK Pathway. Exp. Neurol. 310, 14–21. 10.1016/j.expneurol.2018.08.007 30138606

[B226] Perea J. R. Bolós M. Cuadros R. García E. García-Escudero V. Hernández F. (2022). p38 Inhibition Decreases Tau Toxicity in Microglia and Improves Their Phagocytic Function. Mol. Neurobiol. 59, 1632–1648. 10.1007/s12035-021-02715-0 35006531 PMC8882095

[B227] Perez Ortiz J. M. Swerdlow R. H. (2019). Mitochondrial Dysfunction in Alzheimer's Disease: Role in Pathogenesis and Novel Therapeutic Opportunities. Br. J. Pharmacol. 176, 3489–3507. 10.1111/bph.14585 30675901 PMC6715612

[B228] Perrin R. J. Fagan A. M. Holtzman D. M. (2009). Multimodal Techniques for Diagnosis and Prognosis of Alzheimer's Disease. Nature 461, 916–922. 10.1038/nature08538 19829371 PMC2810658

[B229] Pervin M. Unno K. Nakagawa A. Takahashi Y. Iguchi K. Yamamoto H. (2017). Blood Brain Barrier Permeability of (-)-epigallocatechin Gallate, its Proliferation-Enhancing Activity of Human Neuroblastoma SH-Sy5y Cells, and its Preventive Effect on Age-Related Cognitive Dysfunction in Mice. Biochem. Biophys. Rep. 9, 180–186. 10.1016/j.bbrep.2016.12.012 28956003 PMC5614586

[B230] Pervin M. Unno K. Takagaki A. Isemura M. Nakamura Y. (2019). Function of Green Tea Catechins in the Brain: Epigallocatechin Gallate and its Metabolites. Int. J. Mol. Sci. 20, 3630. 10.3390/ijms20153630 PMC6696481 31349535

[B231] Petrov D. Pedrós I. Artiach G. Sureda F. X. Barroso E. Pallàs M. (2015). High-fat Diet-Induced Deregulation of Hippocampal Insulin Signaling and Mitochondrial Homeostasis Deficiences Contribute to Alzheimer Disease Pathology in Rodents. Biochim. Biophys. Acta 1852, 1687–1699. 10.1016/j.bbadis.2015.05.004 26003667

[B232] Piccialli I. Tedeschi V. Boscia F. Ciccone R. Casamassa A. de Rosa V. (2020). The *Anemonia Sulcata* Toxin BDS-I Protects Astrocytes Exposed to Aβ_1-42_ Oligomers by Restoring [Ca^2+^]_i_ Transients and ER Ca^2+^ Signaling. Toxins (Basel) 13 (1), 20. 10.3390/toxins13010020 33396295 PMC7823622

[B233] Piccialli I. Tedeschi V. Caputo L. Amato G. De Martino L. De Feo V. (2021). The Antioxidant Activity of Limonene Counteracts Neurotoxicity Triggered byAβ1-42 Oligomers in Primary Cortical Neurons. Antioxidants (Basel) 10, 937. 10.3390/antiox10060937 34207788 PMC8227170

[B234] Piccinelli A. C. Morato P. N. Dos Santos Barbosa M. Croda J. Sampson J. Kong X. (2017). Limonene Reduces Hyperalgesia Induced by Gp120 and Cytokines by Modulation of IL-1 β and Protein Expression in Spinal Cord of Mice. Life Sci. 174, 28–34. 10.1016/j.lfs.2016.11.017 27888114

[B235] Planel E. Tatebayashi Y. Miyasaka T. Liu L. Wang L. Herman M. (2007). Insulin Dysfunction Induces *In Vivo* Tau Hyperphosphorylation through Distinct Mechanisms. J. Neurosci. 27, 13635–13648. 10.1523/JNEUROSCI.3949-07.2007 18077675 PMC6673606

[B236] Porro C. Cianciulli A. Trotta T. Lofrumento D. D. Panaro M. A. (2019). Curcumin Regulates Anti-inflammatory Responses by JAK/STAT/SOCS Signaling Pathway in BV-2 Microglial Cells. Biology (Basel) 8, 51. 10.3390/biology8030051 PMC6784227 31252572

[B237] Potter K. A. Buck A. C. Self W. K. Callanan M. E. Sunil S. Capadona J. R. (2013). The Effect of Resveratrol on Neurodegeneration and Blood Brain Barrier Stability Surrounding Intracortical Microelectrodes. Biomaterials 34, 7001–7015. 10.1016/j.biomaterials.2013.05.035 23791503

[B238] Priller C. Bauer T. Mitteregger G. Krebs B. Kretzschmar H. A. Herms J. (2006). Synapse Formation and Function Is Modulated by the Amyloid Precursor Protein. J. Neurosci. 26, 7212–7221. 10.1523/JNEUROSCI.1450-06.2006 16822978 PMC6673945

[B239] Qiang X. Sang Z. Yuan W. Li Y. Liu Q. Bai P. (2014). Design, Synthesis and Evaluation of Genistein-O-Alkylbenzylamines as Potential Multifunctional Agents for the Treatment of Alzheimer's Disease. Eur. J. Med. Chem. 76, 314–331. 10.1016/j.ejmech.2014.02.045 24589487

[B240] Querfurth H. W. LaFerla F. M. (2010). Alzheimer's Disease. N. Engl. J. Med. 362, 329–344. 10.1056/NEJMra0909142 20107219

[B242] Rahman M. H. Bajgai J. Fadriquela A. Sharma S. Trinh T. T. Akter R. (2021). Therapeutic Potential of Natural Products in Treating Neurodegenerative Disorders and Their Future Prospects and Challenges. Molecules 26, 5327. 10.3390/molecules26175327 34500759 PMC8433718

[B243] Ramsey C. P. Glass C. A. Montgomery M. B. Lindl K. A. Ritson G. P. Chia L. A. (2007). Expression of Nrf2 in Neurodegenerative Diseases. J. Neuropathol. Exp. Neurol. 66, 75–85. 10.1097/nen.0b013e31802d6da9 17204939 PMC2253896

[B244] Raposo D. Morgado C. Pereira-Terra P. Tavares I. (2015). Nociceptive Spinal Cord Neurons of Laminae I-III Exhibit Oxidative Stress Damage during Diabetic Neuropathy Which Is Prevented by Early Antioxidant Treatment with Epigallocatechin-Gallate (EGCG). Brain Res. Bull. 110, 68–75. 10.1016/j.brainresbull.2014.12.004 25522867

[B245] Rehman M. U. Tahir M. Khan A. Q. Khan R. Oday-O-Hamiza Lateef A. (2014). D-limonene Suppresses Doxorubicin-Induced Oxidative Stress and Inflammation via Repression of COX-2, iNOS, and NFκB in Kidneys of Wistar Rats. Exp. Biol. Med. (Maywood) 239, 465–476. 10.1177/1535370213520112 24586096

[B246] Reiber H. Ruff M. Uhr M. (1993). Ascorbate Concentration in Human Cerebrospinal Fluid (CSF) and Serum. Intrathecal Accumulation and CSF Flow Rate. Clin. Chim. Acta 217, 163–173. 10.1016/0009-8981(93)90162-w 8261625

[B247] Reno C. M. Puente E. C. Sheng Z. Daphna-Iken D. Bree A. J. Routh V. H. (2017). Brain GLUT4 Knockout Mice Have Impaired Glucose Tolerance, Decreased Insulin Sensitivity, and Impaired Hypoglycemic Counterregulation. Diabetes 66, 587–597. 10.2337/db16-0917 27797912 PMC5319720

[B248] Rezai-Zadeh K. Shytle D. Sun N. Mori T. Hou H. Jeanniton D. (2005). Green tea Epigallocatechin-3-Gallate (EGCG) Modulates Amyloid Precursor Protein Cleavage and Reduces Cerebral Amyloidosis in Alzheimer Transgenic Mice. J. Neurosci. 25, 8807–8814. 10.1523/JNEUROSCI.1521-05.2005 16177050 PMC6725500

[B249] Ries M. Sastre M. (2016). Mechanisms of Aβ Clearance and Degradation by Glial Cells. Front. Aging Neurosci. 8, 160. 10.3389/fnagi.2016.00160 27458370 PMC4932097

[B250] Ringman J. M. Frautschy S. A. Teng E. Begum A. N. Bardens J. Beigi M. (2012). Oral Curcumin for Alzheimer's Disease: Tolerability and Efficacy in a 24-week Randomized, Double Blind, Placebo-Controlled Study. Alzheimers. Res. Ther. 4, 43. 10.1186/alzrt146 PMC3580400 23107780

[B251] Roberto D. Micucci P. Sebastian T. Graciela F. Anesini C. (2010). Antioxidant Activity of Limonene on normal Murine Lymphocytes: Relation to H2O2 Modulation and Cell Proliferation. Basic Clin. Pharmacol. Toxicol. 106, 38–44. 10.1111/j.1742-7843.2009.00467.x 19796276

[B252] Sabogal-Guáqueta A. M. Hobbie F. Keerthi A. Oun A. Kortholt A. Boddeke E. (2019). Linalool Attenuates Oxidative Stress and Mitochondrial Dysfunction Mediated by Glutamate and NMDA Toxicity. Biomed. Pharmacother. 118, 109295. 10.1016/j.biopha.2019.109295 31545255

[B253] Sabogal-Guáqueta A. M. Muñoz-Manco J. I. Ramírez-Pineda J. R. Lamprea-Rodriguez M. Osorio E. Cardona-Gómez G. P. (2015). The Flavonoid Quercetin Ameliorates Alzheimer's Disease Pathology and Protects Cognitive and Emotional Function in Aged Triple Transgenic Alzheimer's Disease Model Mice. Neuropharmacology 93, 134–145. 10.1016/j.neuropharm.2015.01.027 25666032 PMC4387064

[B254] Sabogal-Guáqueta A. M. Osorio E. Cardona-Gómez G. P. (2016). Linalool Reverses Neuropathological and Behavioral Impairments in Old Triple Transgenic Alzheimer's Mice. Neuropharmacology 102, 111–120. 10.1016/j.neuropharm.2015.11.002 26549854 PMC4698173

[B255] Sahara N. Murayama M. Lee B. Park J. M. Lagalwar S. Binder L. I. (2008). Active C-Jun N-Terminal Kinase Induces Caspase Cleavage of Tau and Additional Phosphorylation by GSK-3beta Is Required for Tau Aggregation. Eur. J. Neurosci. 27, 2897–2906. 10.1111/j.1460-9568.2008.06258.x 18540881

[B256] Santiago J. A. Potashkin J. A. (2021). The Impact of Disease Comorbidities in Alzheimer's Disease. Front. Aging Neurosci. 13, 631770. 10.3389/fnagi.2021.631770 33643025 PMC7906983

[B257] Scala I. Valenti D. Scotto D'Aniello V. Marino M. Riccio M. P. Bravaccio C. (2021). Epigallocatechin-3-Gallate Plus Omega-3 Restores the Mitochondrial Complex I and F0F1-ATP Synthase Activities in PBMCs of Young Children with Down Syndrome: A Pilot Study of Safety and Efficacy. Antioxidants (Basel) 10, 469. 10.3390/antiox10030469 33809669 PMC8002266

[B258] Schroeder E. K. Kelsey N. A. Doyle J. Breed E. Bouchard R. J. Loucks F. A. (2009). Green tea Epigallocatechin 3-gallate Accumulates in Mitochondria and Displays a Selective Antiapoptotic Effect against Inducers of Mitochondrial Oxidative Stress in Neurons. Antioxid. Redox Signal. 11, 469–480. 10.1089/ars.2008.2215 18754708 PMC13148728

[B259] Selkoe D. J. (2002). Deciphering the Genesis and Fate of Amyloid Beta-Protein Yields Novel Therapies for Alzheimer Disease. J. Clin. Invest. 110, 1375–1381. 10.1172/JCI16783 12438432 PMC151820

[B260] Selkoe D. J. Hardy J. (2016). The Amyloid Hypothesis of Alzheimer's Disease at 25 Years. EMBO Mol. Med. 8, 595–608. 10.15252/emmm.201606210 27025652 PMC4888851

[B261] Selkoe D. J. (1991). The Molecular Pathology of Alzheimer's Disease. Neuron 6, 487–498. 10.1016/0896-6273(91)90052-2 1673054

[B262] Sengupta U. Nilson A. N. Kayed R. (2016). The Role of Amyloid-β Oligomers in Toxicity, Propagation, and Immunotherapy. EBioMedicine 6, 42–49. 10.1016/j.ebiom.2016.03.035 27211547 PMC4856795

[B263] Seo E. J. Fischer N. Efferth T. (2018). Phytochemicals as Inhibitors of NF-Κb for Treatment of Alzheimer's Disease. Pharmacol. Res. 129, 262–273. 10.1016/j.phrs.2017.11.030 29179999

[B264] Seong K. J. Lee H. G. Kook M. S. Ko H. M. Jung J. Y. Kim W. J. (2016). Epigallocatechin-3-gallate Rescues LPS-Impaired Adult Hippocampal Neurogenesis through Suppressing the TLR4-NF-Κb Signaling Pathway in Mice. Korean J. Physiol. Pharmacol. 20, 41–51. 10.4196/kjpp.2016.20.1.41 26807022 PMC4722190

[B265] Serrano-Pozo A. Qian J. Monsell S. E. Blacker D. Gómez-Isla T. Betensky R. A. (2014). Mild to Moderate Alzheimer Dementia with Insufficient Neuropathological Changes. Ann. Neurol. 75, 597–601. 10.1002/ana.24125 24585367 PMC4016558

[B266] Serretti A. Olgiati P. De Ronchi D. (2007). Genetics of Alzheimer's Disease. A Rapidly Evolving Field. J. Alzheimers Dis. 12, 73–92. 10.3233/jad-2007-12108 17851196

[B267] Seubert P. Oltersdorf T. Lee M. G. Barbour R. Blomquist C. Davis D. L. (1993). Secretion of Beta-Amyloid Precursor Protein Cleaved at the Amino Terminus of the Beta-Amyloid Peptide. Nature 361, 260–263. 10.1038/361260a0 7678698

[B268] Shabir O. Berwick J. Francis S. E. (2018). Neurovascular Dysfunction in Vascular Dementia, Alzheimer's and Atherosclerosis. BMC Neurosci. 19, 62. 10.1186/s12868-018-0465-5 30333009 PMC6192291

[B269] Shahbaz S. K. Koushki K. Sathyapalan T. Majeed M. Sahebkar A. (2021). PLGA-based Curcumin Delivery System: An Interesting Therapeutic Approach in Treatment of Alzheimer's Disease. Curr. Neuropharmacol. 20, 309–323. 10.2174/1570159X19666210823103020 PMC9413791 34429054

[B270] Sharma C. Kim S. R. (2021). Linking Oxidative Stress and Proteinopathy in Alzheimer's Disease. Antioxidants (Basel) 10, 1231. 10.3390/antiox10081231 34439479 PMC8388980

[B271] Sharma V. Mishra M. Ghosh S. Tewari R. Basu A. Seth P. (2007). Modulation of Interleukin-1beta Mediated Inflammatory Response in Human Astrocytes by Flavonoids: Implications in Neuroprotection. Brain Res. Bull. 73, 55–63. 10.1016/j.brainresbull.2007.01.016 17499637

[B272] Shi X. Zheng Z. Li J. Xiao Z. Qi W. Zhang A. (2015). Curcumin Inhibits Aβ-Induced Microglial Inflammatory Responses *In Vitro*: Involvement of ERK1/2 and P38 Signaling Pathways. Neurosci. Lett. 594, 105–110. 10.1016/j.neulet.2015.03.045 25818332

[B273] Shi Y. Andhey P. S. Ising C. Wang K. Snipes L. L. Boyer K. (2021). Overexpressing Low-Density Lipoprotein Receptor Reduces Tau-Associated Neurodegeneration in Relation to apoE-Linked Mechanisms. Neuron 109, 2413–2426. e7. 10.1016/j.neuron.2021.05.034 34157306 PMC8349883

[B274] Shi Y. Yamada K. Liddelow S. A. Smith S. T. Zhao L. Luo W. (2017). ApoE4 Markedly Exacerbates Tau-Mediated Neurodegeneration in a Mouse Model of Tauopathy. Nature 549, 523–527. 10.1038/nature24016 28959956 PMC5641217

[B275] Shin M. Liu Q. F. Choi B. Shin C. Lee B. Yuan C. (2020). Neuroprotective Effects of Limonene (+) against Aβ42-Induced Neurotoxicity in a Drosophila Model of Alzheimer's Disease. Biol. Pharm. Bull. 43, 409–417. 10.1248/bpb.b19-00495 31875578

[B276] Singh G. Pachouri U. C. Khaidem D. C. Kundu A. Chopra C. Singh P. (2015). Mitochondrial DNA Damage and Diseases. F1000Research 4, 176. 10.12688/f1000research.6665.1 27508052 PMC4962287

[B277] Siracusa R. Impellizzeri D. Cordaro M. Crupi R. Esposito E. Petrosino S. (2017). Anti-Inflammatory and Neuroprotective Effects of Co-UltraPEALut in a Mouse Model of Vascular Dementia. Front. Neurol. 8, 233. 10.3389/fneur.2017.00233 28634464 PMC5460147

[B278] Snyder E. M. Nong Y. Almeida C. G. Paul S. Moran T. Choi E. Y. (2005). Regulation of NMDA Receptor Trafficking by Amyloid-Beta. Nat. Neurosci. 8, 1051–1058. 10.1038/nn1503 16025111

[B279] Solfrizzi V. D'Introno A. Colacicco A. M. Capurso C. Todarello O. Pellicani V. (2006). Circulating Biomarkers of Cognitive Decline and Dementia. Clin. Chim. Acta 364, 91–112. 10.1016/j.cca.2005.06.015 16139826

[B280] Song T. Song X. Zhu C. Patrick R. Skurla M. Santangelo I. (2021). Mitochondrial Dysfunction, Oxidative Stress, Neuroinflammation, and Metabolic Alterations in the Progression of Alzheimer's Disease: A Meta-Analysis of *In Vivo* Magnetic Resonance Spectroscopy Studies. Ageing Res. Rev. 72, 101503. 10.1016/j.arr.2021.101503 34751136 PMC8662951

[B281] Sorrenti V. Contarini G. Sut S. Dall'Acqua S. Confortin F. Pagetta A. (2018). Curcumin Prevents Acute Neuroinflammation and Long-Term Memory Impairment Induced by Systemic Lipopolysaccharide in Mice. Front. Pharmacol. 9, 183. 10.3389/fphar.2018.00183 29556196 PMC5845393

[B282] Soto-Rojas L. O. Campa-Córdoba B. B. Harrington C. R. Salas-Casas A. Hernandes-Alejandro M. Villanueva-Fierro I. (2021). Insoluble Vascular Amyloid Deposits Trigger Disruption of the Neurovascular Unit in Alzheimer's Disease Brains. Int. J. Mol. Sci. 22, 3654. 10.3390/ijms22073654 33915754 PMC8036769

[B283] Spagnuolo C. Moccia S. Russo G. L. (2018). Anti-inflammatory Effects of Flavonoids in Neurodegenerative Disorders. Eur. J. Med. Chem. 153, 105–115. 10.1016/j.ejmech.2017.09.001 28923363

[B284] Spencer J. P. Vafeiadou K. Williams R. J. Vauzour D. (2012). Neuroinflammation: Modulation by Flavonoids and Mechanisms of Action. Mol. Aspects Med. 33, 83–97. 10.1016/j.mam.2011.10.016 22107709

[B285] Spilsbury A. Vauzour D. Spencer J. P. Rattray M. (2012). Regulation of NF-Κb Activity in Astrocytes: Effects of Flavonoids at Dietary-Relevant Concentrations. Biochem. Biophys. Res. Commun. 418, 578–583. 10.1016/j.bbrc.2012.01.081 22293195

[B286] Srinivasan P. Vijayakumar S. Kothandaraman S. Palani M. (2018). Anti-diabetic Activity of Quercetin Extracted from Phyllanthus Emblica L. Fruit: In Silico and *In Vivo* Approaches. J. Pharm. Anal. 8, 109–118. 10.1016/j.jpha.2017.10.005 29736297 PMC5934737

[B287] Sripetchwandee J. Chattipakorn N. Chattipakorn S. C. (2018). Links between Obesity-Induced Brain Insulin Resistance, Brain Mitochondrial Dysfunction, and Dementia. Front. Endocrinol. (Lausanne) 9, 496. 10.3389/fendo.2018.00496 30233495 PMC6127253

[B288] Steen E. Terry B. M. Rivera E. J. Cannon J. L. Neely T. R. Tavares R. (2005). Impaired Insulin and Insulin-like Growth Factor Expression and Signaling Mechanisms in Alzheimer’s Disease - Is This Type 3 Diabetes? J. Alzheimers Dis. 7, 63–80. 10.3233/jad-2005-7107 15750215

[B289] Stefanescu R. Stanciu G. D. Luca A. Paduraru L. Tamba B. I. (2020). Secondary Metabolites from Plants Possessing Inhibitory Properties against Beta-Amyloid Aggregation as Revealed by Thioflavin-T Assay and Correlations with Investigations on Transgenic Mouse Models of Alzheimer's Disease. Biomolecules 10, 870. 10.3390/biom10060870 PMC7355648 32517180

[B290] Sun P. Chen J. Y. Li J. Sun M. R. Mo W. C. Liu K. L. (2013). The Protective Effect of Geniposide on Human Neuroblastoma Cells in the Presence of Formaldehyde. BMC Complement. Altern. Med. 13, 152. 10.1186/1472-6882-13-152 23815892 PMC3702466

[B291] Sun P. Ding H. Liang M. Li X. Mo W. Wang X. (2014). Neuroprotective Effects of Geniposide in SH-Sy5y Cells and Primary Hippocampal Neurons Exposed to Aβ42. Biomed. Res. Int. 2014, 284314. 10.1155/2014/284314 25506055 PMC4255058

[B292] Szkudelski T. Szkudelska K. (2011). Anti-diabetic Effects of Resveratrol. Ann. N. Y. Acad. Sci. 1215, 34–39. 10.1111/j.1749-6632.2010.05844.x 21261639

[B293] Szwajgier D. Baranowska-Wójcik E. (2019). Terpenes and Phenylpropanoids as Acetyl- and Butyrylcholinesterase Inhibitors: A Comparative Study. Curr. Alzheimer Res. 16, 963–973. 10.2174/1567205016666191010105115 31660828

[B294] Tamagno E. Bardini P. Obbili A. Vitali A. Borghi R. Zaccheo D. (2002). Oxidative Stress Increases Expression and Activity of BACE in NT2 Neurons. Neurobiol. Dis. 10, 279–288. 10.1006/nbdi.2002.0515 12270690

[B295] Taylor M. Moore S. Mourtas S. Niarakis A. Re F. Zona C. (2011). Effect of Curcumin-Associated and Lipid Ligand-Functionalized Nanoliposomes on Aggregation of the Alzheimer's Aβ Peptide. Nanomedicine 7, 541–550. 10.1016/j.nano.2011.06.015 21722618

[B296] Terzo S. Amato A. Mulè F. (2021). From Obesity to Alzheimer's Disease through Insulin Resistance. J. Diabetes Complications 35, 108026. 10.1016/j.jdiacomp.2021.108026 34454830

[B297] Tian Y. Ma J. Wang W. Zhang L. Xu J. Wang K. (2016b). Resveratrol Supplement Inhibited the NF-Κb Inflammation Pathway through Activating AMPKα-SIRT1 Pathway in Mice with Fatty Liver. Mol. Cel. Biochem. 422, 75–84. 10.1007/s11010-016-2807-x 27613163

[B298] Tian Y. Y. Jiang B. An L. J. Bao Y. M. (2007). Neuroprotective Effect of Catalpol against MPP(+)-induced Oxidative Stress in Mesencephalic Neurons. Eur. J. Pharmacol. 568, 142–148. 10.1016/j.ejphar.2007.04.039 17512520

[B299] Tian Z. Wang J. Xu M. Wang Y. Zhang M. Zhou Y. (2016a). Resveratrol Improves Cognitive Impairment by Regulating Apoptosis and Synaptic Plasticity in Streptozotocin-Induced Diabetic Rats. Cell. Physiol. Biochem. 40, 1670–1677. 10.1159/000453216 28006780

[B300] Tomaselli S. La Vitola P. Pagano K. Brandi E. Santamaria G. Galante D. (2019). Biophysical and *In Vivo* Studies Identify a New Natural-Based Polyphenol, Counteracting Aβ Oligomerization *In Vitro* and Aβ Oligomer-Mediated Memory Impairment and Neuroinflammation in an Acute Mouse Model of Alzheimer's Disease. ACS Chem. Neurosci. 10, 4462–4475. 10.1021/acschemneuro.9b00241 31603646

[B301] Tong Y. Zhou W. Fung V. Christensen M. A. Qing H. Sun X. (2005). Oxidative Stress Potentiates BACE1 Gene Expression and Abeta Generation. J. Neural Transm. (Vienna) 112, 455–469. 10.1007/s00702-004-0255-3 15614428

[B302] Tozaki H. Matsumoto A. Kanno T. Nagai K. Nagata T. Yamamoto S. (2002). The Inhibitory and Facilitatory Actions of Amyloid-Beta Peptides on Nicotinic ACh Receptors and AMPA Receptors. Biochem. Biophys. Res. Commun. 294, 42–45. 10.1016/S0006-291X(02)00429-1 12054737

[B303] Trejo-Lopez J. A. Yachnis A. T. Prokop S. (2021). Neuropathology of Alzheimer's Disease. Neurotherapeutics 2021. 10.1007/s13311-021-01146-y PMC9130398 34729690

[B304] Tsunoda T. Takase M. Shigemori H. (2018). Structure-Activity Relationship of Clovamide and its Related Compounds for the Inhibition of Amyloid β Aggregation. Bioorg. Med. Chem. 26, 3202–3209. 10.1016/j.bmc.2018.04.044 29706525

[B305] Uddin M. S. Hasana S. Ahmad J. Hossain M. F. Rahman M. M. Behl T. (2021). Anti-Neuroinflammatory Potential of Polyphenols by Inhibiting NF-Κb to Halt Alzheimer's Disease. Curr. Pharm. Des. 27, 402–414. 10.2174/1381612826666201118092422 33213314

[B306] Valles S. L. Dolz-Gaiton P. Gambini J. Borras C. LLoret A. Pallardo F. V. (2010). Estradiol or Genistein Prevent Alzheimer's Disease-Associated Inflammation Correlating with an Increase PPAR Gamma Expression in Cultured Astrocytes. Brain Res. 1312, 138–144. 10.1016/j.brainres.2009.11.044 19948157

[B307] van de Haar H. J. Burgmans S. Jansen J. F. van Osch M. J. van Buchem M. A. Muller M. (2016). Blood-Brain Barrier Leakage in Patients with Early Alzheimer Disease. Radiology 281, 527–535. 10.1148/radiol.2016152244 27243267

[B308] van der Merwe M. (2021). Gut Microbiome Changes Induced by a Diet Rich in Fruits and Vegetables. Int. J. Food Sci. Nutr. 72, 665–669. 10.1080/09637486.2020.1852537 33960869

[B309] Verkhratsky A. Rodríguez-Arellano J. J. Parpura V. Zorec R. (2017). Astroglial Calcium Signalling in Alzheimer's Disease. Biochem. Biophys. Res. Commun. 483, 1005–1012. 10.1016/j.bbrc.2016.08.088 27545605

[B310] Vesa C. M. Popa L. Popa A. R. Rus M. Zaha A. A. Bungau S. (2020). Current Data Regarding the Relationship between Type 2 Diabetes Mellitus and Cardiovascular Risk Factors. Diagnostics (Basel) 10, 314. 10.3390/diagnostics10050314 PMC7277953 32429441

[B311] Walia V. Kaushik D. Mittal V. Kumar K. Verma R. Parashar J. (2021). Delineation of Neuroprotective Effects and Possible Benefits of AntioxidantsTherapy for the Treatment of Alzheimer's Diseases by Targeting Mitochondrial-Derived Reactive Oxygen Species: Bench to Bedside. Mol. Neurobiol. 59, 657–680. 10.1007/s12035-021-02617-1 34751889

[B312] Wang F. Cui N. Yang L. Shi L. Li Q. Zhang G. (2015). Resveratrol Rescues the Impairments of Hippocampal Neurons Stimulated by Microglial Over-activation *In Vitro* . Cell. Mol. Neurobiol. 35, 1003–1015. 10.1007/s10571-015-0195-5 25898934 PMC11486292

[B313] Wang J. Z. Xia Y. Y. Grundke-Iqbal I. Iqbal K. (2013). Abnormal Hyperphosphorylation of Tau: Sites, Regulation, and Molecular Mechanism of Neurofibrillary Degeneration. J. Alzheimers Dis. 33 (Suppl. 1), S123–S139. 10.3233/JAD-2012-129031 22710920

[B314] Wang Y. Cai B. Shao J. Wang T. Cai R. Ma C. (2016). Genistein Suppresses the Mitochondrial Apoptotic Pathway in Hippocampal Neurons in Rats with Alzheimer's Disease. Neural Regen. Res. 11, 1153–1158. 10.4103/1673-5374.187056 27630702 PMC4994461

[B315] Wang Z. Huang X. Zhao P. Zhao L. Wang Z. Y. (2018). Catalpol Inhibits Amyloid-β Generation through Promoting α-Cleavage of APP in Swedish Mutant APP Overexpressed N2a Cells. Front. Aging Neurosci. 10, 66. 10.3389/fnagi.2018.00066 PMC5867310 29615891

[B316] Wei Z. Koya J. Reznik S. E. (2021). Insulin Resistance Exacerbates Alzheimer Disease via Multiple Mechanisms. Front. Neurosci. 15, 687157. 10.3389/fnins.2021.687157 34349617 PMC8326507

[B317] Westin K. Buchhave P. Nielsen H. Minthon L. Janciauskiene S. Hansson O. (2012). CCL2 Is Associated with a Faster Rate of Cognitive Decline during Early Stages of Alzheimer's Disease. PLoS One 7, e30525. 10.1371/journal.pone.0030525 22303443 PMC3268759

[B318] Wojtunik-Kulesza K. A. Kasprzak K. Oniszczuk T. Oniszczuk A. (2019). Natural Monoterpenes: Much More Than Only a Scent. Chem. Biodivers. 16, e1900434. 10.1002/cbdv.201900434 31587473

[B319] Woodburn S. C. Bollinger J. L. Wohleb E. S. (2021). The Semantics of Microglia Activation: Neuroinflammation, Homeostasis, and Stress. J. Neuroinflammation 18, 258. 10.1186/s12974-021-02309-6 34742308 PMC8571840

[B320] Xu F. Fu Z. Dass S. Kotarba A. E. Davis J. Smith S. O. (2016). Cerebral Vascular Amyloid Seeds Drive Amyloid β-protein Fibril Assembly with a Distinct Anti-parallel Structure. Nat. Commun. 7, 13527. 10.1038/ncomms13527 27869115 PMC5121328

[B321] Xu N. Zhang L. Dong J. Zhang X. Chen Y. G. Bao B. (2014). Low-dose Diet Supplement of a Natural Flavonoid, Luteolin, Ameliorates Diet-Induced Obesity and Insulin Resistance in Mice. Mol. Nutr. Food Res. 58, 1258–1268. 10.1002/mnfr.201300830 24668788

[B322] Xu P. Wang K. Lu C. Dong L. Gao L. Yan M. (2017). The Protective Effect of Lavender Essential Oil and its Main Component Linalool against the Cognitive Deficits Induced by D-Galactose and Aluminum Trichloride in Mice. Evid. Based Complement. Alternat. Med. 2017, 7426538. 10.1155/2017/7426538 28529531 PMC5424179

[B323] Xue Q. Liu Y. He R. Yang S. Tong J. Li X. (2016). Lyophilized Powder of Catalpol and Puerarin Protects Neurovascular Unit from Stroke. Int. J. Biol. Sci. 12, 367–380. 10.1007/s12035-021-02389-810.7150/ijbs.14059 27019622 PMC4807157

[B324] Yamazaki M. Sakura N. Chiba K. Mohri T. (2001). Prevention of the Neurotoxicity of the Amyloid Beta Protein by Genipin. Biol. Pharm. Bull. 24, 1454–1455. 10.1248/bpb.24.1454 11767124

[B325] Yan J. Wang C. Jin Y. Meng Q. Liu Q. Liu Z. (2018). Catalpol Ameliorates Hepatic Insulin Resistance in Type 2 Diabetes through Acting on AMPK/NOX4/PI3K/AKT Pathway. Pharmacol. Res. 130, 466–480. 10.1016/j.phrs.2017.12.026 29284152

[B326] Yanagisawa D. Amatsubo T. Morikawa S. Taguchi H. Urushitani M. Shirai N. (2011). *In Vivo* detection of Amyloid β Deposition Using 19F Magnetic Resonance Imaging with a 19F-Containing Curcumin Derivative in a Mouse Model of Alzheimer's Disease. Neuroscience 184, 120–127. 10.1016/j.neuroscience.2011.03.071 21497641

[B327] Yang D. K. Kang H. S. (2018). Anti-Diabetic Effect of Cotreatment with Quercetin and Resveratrol in Streptozotocin-Induced Diabetic Rats. Biomol. Ther. (Seoul) 26, 130–138. 10.4062/biomolther.2017.254 29462848 PMC5839491

[B328] Yang F. Lim G. P. Begum A. N. Ubeda O. J. Simmons M. R. Ambegaokar S. S. (2005). Curcumin Inhibits Formation of Amyloid Beta Oligomers and Fibrils, Binds Plaques, and Reduces Amyloid *In Vivo* . J. Biol. Chem. 280, 5892–5901. 10.1074/jbc.M404751200 15590663

[B329] Yang T. Li S. Xu H. Walsh D. M. Selkoe D. J. (2017). Large Soluble Oligomers of Amyloid β-Protein from Alzheimer Brain Are Far Less Neuroactive Than the Smaller Oligomers to Which They Dissociate. J. Neurosci. 37, 152–163. 10.1523/JNEUROSCI.1698-16.2016 28053038 PMC5214627

[B330] Yao Y. Li J. Niu Y. Yu J. Q. Yan L. Miao Z. H. (2015). Resveratrol Inhibits Oligomeric Aβ-Induced Microglial Activation via NADPH Oxidase. Mol. Med. Rep. 12, 6133–6139. 10.3892/mmr.2015.4199 26252250

[B331] Yin F. Liu J. H. Zheng X. X. Guo L. X. (2010b). GLP-1 Receptor Plays a Critical Role in Geniposide-Induced Expression of Heme Oxygenase-1 in PC12 Cells. Acta Pharmacol. Sin. 31, 540–545. 10.1038/aps.2010.28 20364157 PMC4002743

[B332] Yin F. Liu J. Zheng X. Guo L. Xiao H. (2010a). Geniposide Induces the Expression of Heme Oxygenase-1 via PI3K/Nrf2-Signaling to Enhance the Antioxidant Capacity in Primary Hippocampal Neurons. Biol. Pharm. Bull. 33, 1841–1846. 10.1248/bpb.33.1841 21048309

[B333] Yin F. Zhang Y. Guo L. Kong S. Liu J. (2012). Geniposide Regulates Insulin-Degrading Enzyme Expression to Inhibit the Cytotoxicity of Aβ₁₋₄₂ in Cortical Neurons. CNS Neurol. Disord. Drug Targets 11, 1045–1051. 10.2174/1871527311211080015 23244428

[B334] Yoon S. S. Jo S. A. (2012). Mechanisms of Amyloid-β Peptide Clearance: Potential Therapeutic Targets for Alzheimer's Disease. Biomol. Ther. (Seoul) 20, 245–255. 10.4062/biomolther.2012.20.3.245 24130920 PMC3794520

[B335] Youn K. Park J. H. Lee S. Lee S. Lee J. Yun E. Y. (2018). BACE1 Inhibition by Genistein: Biological Evaluation, Kinetic Analysis, and Molecular Docking Simulation. J. Med. Food 21, 416–420. 10.1089/jmf.2017.4068 29444415

[B336] Yu C. Nwabuisi-Heath E. Laxton K. Ladu M. J. (2010). Endocytic Pathways Mediating Oligomeric Abeta42 Neurotoxicity. Mol. Neurodegen. 5, 19. 10.1186/1750-1326-5-19 PMC2881055 20478062

[B337] Yu X. Li Y. Mu X. (2020). Effect of Quercetin on PC12 Alzheimer's Disease Cell Model Induced by Aβ25-35 and its Mechanism Based on Sirtuin1/Nrf2/HO-1 Pathway. Biomed. Res. Int. 2020, 8210578. 10.1155/2020/8210578 32420373 PMC7201675

[B338] Zatterale F. Longo M. Naderi J. Raciti G. A. Desiderio A. Miele C. (2020). Chronic Adipose Tissue Inflammation Linking Obesity to Insulin Resistance and Type 2 Diabetes. Front. Physiol. 10, 1607. 10.3389/fphys.2019.01607 32063863 PMC7000657

[B339] Zhan C. Chen Y. Tang Y. Wei G. (2020). Green Tea Extracts EGCG and EGC Display Distinct Mechanisms in Disrupting Aβ42 Protofibril. ACS Chem. Neurosci. 11, 1841–1851. 10.1021/acschemneuro.0c00277 32441920

[B340] Zhang A. Hao S. Bi J. Bao Y. Zhang X. An L. (2009). Effects of Catalpol on Mitochondrial Function and Working Memory in Mice after Lipopolysaccharide-Induced Acute Systemic Inflammation. Exp. Toxicol. Pathol. 61, 461–469. 10.1016/j.etp.2008.10.010 19081713

[B341] Zhang F. Wang H. Wu Q. Lu Y. Nie J. Xie X. (2013). Resveratrol Protects Cortical Neurons against Microglia-Mediated Neuroinflammation. Phytother. Res. 27, 344–349. 10.1002/ptr.4734 22585561

[B342] Zhang H. Zhao C. Lv C. Liu X. Du S. Li Z. (2017). Geniposide Alleviates Amyloid-Induced Synaptic Injury by Protecting Axonal Mitochondrial Trafficking. Front. Cel. Neurosci. 10, 309. 10.3389/fncel.2016.00309 PMC5263130 28179878

[B343] Zhang J. Feng X. Wu J. Xu H. Li G. Zhu D. (2014). Neuroprotective Effects of Resveratrol on Damages of Mouse Cortical Neurons Induced by β-amyloid through Activation of SIRT1/Akt1 Pathway. Biofactors 40, 258–267. 10.1002/biof.1149 24132831

[B344] Zhang J. S. Zhou S. F. Wang Q. Guo J. N. Liang H. M. Deng J. B. (2016). Gastrodin Suppresses BACE1 Expression under Oxidative Stress Condition via Inhibition of the PKR/eIF2α Pathway in Alzheimer's Disease. Neuroscience 325, 1–9. 10.1016/j.neuroscience.2016.03.024 26987953

[B345] Zhang J. Zheng Y. Luo Y. Du Y. Zhang X. Fu J. (2019). Curcumin Inhibits LPS-Induced Neuroinflammation by Promoting Microglial M2 Polarization via TREM2/TLR4/NF-Κb Pathways in BV2 Cells. Mol. Immunol. 116, 29–37. 10.1016/j.molimm.2019.09.020 31590042

[B346] Zhang R. Miller R. G. Madison C. Jin X. Honrada R. Harris W. (2013). Systemic Immune System Alterations in Early Stages of Alzheimer's Disease. J. Neuroimmunol. 256, 38–42. 10.1016/j.jneuroim.2013.01.002 23380586 PMC3641776

[B347] Zhang S. Zhu Q. Chen J. Y. OuYang D. Lu J. H. (2020). The Pharmacological Activity of Epigallocatechin-3-Gallate (EGCG) on Alzheimer's Disease Animal Model: A Systematic Review. Phytomedicine 79, 153316. 10.1016/j.phymed.2020.153316 32942205

[B348] Zhang X. Jin C. Li Y. Guan S. Han F. Zhang S. (2013). Catalpol Improves Cholinergic Function and Reduces Inflammatory Cytokines in the Senescent Mice Induced by D-Galactose. Food Chem. Toxicol. 58, 50–55. 10.1016/j.fct.2013.04.006 23612000

[B349] Zhang X. L. An L. J. Bao Y. M. Wang J. Y. Jiang B. (2008b). D-Galactose Administration Induces Memory Loss and Energy Metabolism Disturbance in Mice: Protective Effects of Catalpol. Food Chem. Toxicol. 46, 2888–2894. 10.1016/j.fct.2008.05.032 18573305

[B350] Zhang X. Liu W. Niu X. An L. (2010). Systemic Administration of Catalpol Prevents D-Galactose Induced Mitochondrial Dysfunction in Mice. Neurosci. Lett. 473, 224–228. 10.1016/j.neulet.2010.02.054 20219628

[B351] Zhang X. L. Jiang B. Li Z. B. Hao S. An L. J. (2007). Catalpol Ameliorates Cognition Deficits and Attenuates Oxidative Damage in the Brain of Senescent Mice Induced by D-Galactose. Pharmacol. Biochem. Behav. 88, 64–72. 10.1016/j.pbb.2007.07.004 17698178

[B352] ZhangX. X.TianY.WangZ. T.MaY. H.TanL.YuJ. T. (2021). The Epidemiology of Alzheimer's Disease Modifiable Risk Factors and Prevention. J. Prev. Alzheimers Dis. 8, 313–321. 10.14283/jpad.2021.15 34101789

[B353] Zhang X. Zhang A. Jiang B. Bao Y. Wang J. An L. (2008a). Further Pharmacological Evidence of the Neuroprotective Effect of Catalpol from Rehmannia Glutinosa. Phytomedicine 15, 484–490. 10.1016/j.phymed.2008.01.001 18281203

[B354] Zhang Y. Huang N. Q. Yan F. Jin H. Zhou S. Y. Shi J. S. (2018). Diabetes Mellitus and Alzheimer's Disease: GSK-3β as a Potential Link. Behav. Brain Res. 339, 57–65. 10.1016/j.bbr.2017.11.015 29158110

[B355] Zhang Y. Yin F. Liu J. Liu Z. Guo L. Xia Z. (2015). Geniposide Attenuates Insulin-Deficiency-Induced Acceleration of β-amyloidosis in an APP/PS1 Transgenic Model of Alzheimer's Disease. Neurochem. Int. 89, 7–16. 10.1016/j.neuint.2015.04.002 25882165

[B356] Zhao C. Lv C. Li H. Du S. Liu X. Li Z. (2016). Geniposide Protects Primary Cortical Neurons against Oligomeric Aβ1-42-Induced Neurotoxicity through a Mitochondrial Pathway. PLoS One 11, e0152551. 10.1371/journal.pone.0152551 27046221 PMC4821580

[B357] Zhao C. Zhang H. Li H. Lv C. Liu X. Li Z. (2017). Geniposide Ameliorates Cognitive Deficits by Attenuating the Cholinergic Defect and Amyloidosis in Middle-Aged Alzheimer Model Mice. Neuropharmacology 116, 18–29. 10.1016/j.neuropharm.2016.12.002 27940040

[B358] Zhao H. F. Li N. Wang Q. Cheng X. J. Li X. M. Liu T. T. (2015). Resveratrol Decreases the Insoluble Aβ1-42 Level in hippocampus and Protects the Integrity of the Blood-Brain Barrier in AD Rats. Neuroscience 310, 641–649. 10.1016/j.neuroscience.2015.10.006 26454022

[B359] Zhao H. Wang Q. Cheng X. Li X. Li N. Liu T. (2018). Inhibitive Effect of Resveratrol on the Inflammation in Cultured Astrocytes and Microglia Induced by Aβ1-42. Neuroscience 379, 390–404. 10.1016/j.neuroscience.2018.03.047 29627302

[B360] Zhao L. Teter B. Morihara T. Lim G. P. Ambegaokar S. S. Ubeda O. J. (2004). Insulin-degrading Enzyme as a Downstream Target of Insulin Receptor Signaling cascade: Implications for Alzheimer's Disease Intervention. J. Neurosci. 24, 11120–11126. 10.1523/JNEUROSCI.2860-04.2004 15590928 PMC6730264

[B361] Zhou J. Xu G. Ma S. Li F. Yuan M. Xu H. (2015). Catalpol Ameliorates High-Fat Diet-Induced Insulin Resistance and Adipose Tissue Inflammation by Suppressing the JNK and NF-Κb Pathways. Biochem. Biophys. Res. Commun. 467, 853–858. 10.1016/j.bbrc.2015.10.054 26474703

[B362] Zhou X. Yuan L. Zhao X. Hou C. Ma W. Yu H. (2014). Genistein Antagonizes Inflammatory Damage Induced by β-amyloid Peptide in Microglia through TLR4 and NF-Κb. Nutrition 30, 90–95. 10.1016/j.nut.2013.06.006 24290604

[B363] Zhu L. H. Bi W. Qi R. B. Wang H. D. Lu D. X. (2011). Luteolin Inhibits Microglial Inflammation and Improves Neuron Survival against Inflammation. Int. J. Neurosci. 121, 329–336. 10.3109/00207454.2011.569040 21631167

[B364] Zlokovic B. V. (2011). Neurovascular Pathways to Neurodegeneration in Alzheimer's Disease and Other Disorders. Nat. Rev. Neurosci. 12, 723–738. 10.1038/nrn3114 22048062 PMC4036520

[B365] Zorov D. B. Juhaszova M. Sollott S. J. (2014). Mitochondrial Reactive Oxygen Species (ROS) and ROS-Induced ROS Release. Physiol. Rev. 94, 909–950. 10.1152/physrev.00026.2013 24987008 PMC4101632

[B366] Zott B. Busche M. A. Sperling R. A. Konnerth A. (2018). What Happens with the Circuit in Alzheimer's Disease in Mice and Humans? Annu. Rev. Neurosci. 41, 277–297. 10.1146/annurev-neuro-080317-061725 29986165 PMC6571139

